# Synthesis, molecular modelling, and antibacterial evaluation of new sulfonamide-dyes based pyrrole compounds

**DOI:** 10.1038/s41598-024-60908-8

**Published:** 2024-05-14

**Authors:** Hatem E. Gaffer, S. A. Mahmoud, M. S. El-Sedik, Tarek Aysha, Mohamed H. Abdel-Rhman, Ehab Abdel-latif

**Affiliations:** 1 Dyeing, Printing, and Auxiliaries Department, National Research Centre, Textile Institute, Giza, Cairo, Egypt; 2 Chemistry Department, Faculty of Science, Mansoura University, Mansoura, Egypt

**Keywords:** Sulfonamide, Synthesis, Docking, DFT calculations, Antimicrobial, 6CLV, Chemical biology, Chemistry

## Abstract

In this study, we synthesized new series of 5-oxo-2-phenyl-4-(arylsulfamoyl)sulphenyl) hydrazono)-4,5-dihydro-*1H*-pyrrole-3-carboxylate hybrids **4a-f** with the goal of overcoming sulfonamide resistance and identifying novel therapeutic candidates by chemical changes. The chemical structures of the synthesized hybrids were established over the spectroscopic tools. The frontier molecular orbitals configuration and energetic possessions of the synthesized compounds were discovered utilizing DFT/B3LYP/6-311++ G** procedure. The 3D plots of both HOMO and LUMO showed comparable configuration of both HOMO and LUMO led to close values of their energies. Amongst the prepared analogues, the sulfonamide hybrids **4a-f**, hybrid **4a** presented potent inhibitory towards *S. typhimurium* with (IZD = 15 mm, MIC = 19.24 µg/mL) and significant inhibition with (IZD = 19 mm, MIC = 11.31 µg/mL) against* E.coli* in contrast to sulfonamide (Sulfamethoxazole) reference Whereas, hybrid **4d** demonstrated potent inhibition with (IZD = 16 mm, MIC = 19.24 µg/mL) against *S. typhimurium* with enhanced inhibition against *E. Coli*, Additionally, the generated sulfonamide analogues’' molecular docking was estimated over (PDB: 3TZF and 6CLV) proteins. Analogue **4e** had the highest documented binding score as soon as linked to the other analogues. The docking consequences were fitting and addressed with the antibacterial valuation.

## Introduction

**Figure 1:**
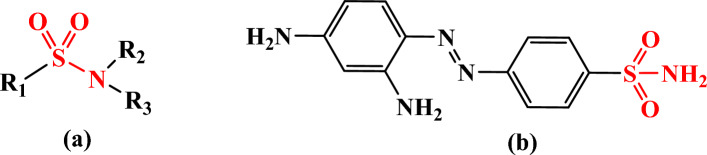
Chemical structure of Sulfonamide skeleton (**a**) and Prontosil red (**b**) (Created by: ChemDraw (Ver. 17)).

Sulfonamides are an antibacterial sulfa-drug. It is an organic molecule made composed of aniline that has been derivatized with a sulfonamide group as in Fig. 1a^1–3^. The Allies in World War II utilized powdered sulfonamide to lower infection rates, which led to a considerable decline in mortality rates compared to previous battles^4,5^. Due to its toxicity and the existence of more efficient sulfonamides, is rarely, if ever, given systemically. Sulfanilamide has been replaced by modern antibiotics on the front lines, but it is still used today in topical treatments for treating vaginal yeast infections, particularly vulvovaginitis, which is brought on by Candida albicans^6,7^. The primary sulfonamide structure, SO_2_NH, is found in a variety of biologically active chemicals that are frequently employed as antibiotics, Anti-hypertension, carbonic anhydrase inhibitors, antithyroid medications, and antimicrobial capsules^8^. Sulfonamides are also very beneficial pharmacological substances because they reveal a wide range of biological properties, such as anti-tumor, anti-inflammatory, and antiviral activity^9–11^. Sulfonamide antibiotics are occasionally used in conjunction with other antibiotics to improve their efficacy^12^. They could be coupled, for example, with trimethoprim, another antibiotic that targets a different phase in the folic acid production pathway^13^. Trimethoprim-sulfamethoxazole, often known as co-trimoxazole, is a popular antibiotic used to treat respiratory, urinary, and gastrointestinal infections. Sulfonamido-chrysoidine (prontosil red, Fig. 1b), one of the azo dyes to cure Streptococcus infection in mice, was published by German bacteriologist and pathologist Gerhard Domagk and proved to be very successful^14^. Sulfonamides are used to treat several gastrointestinal and urinary tract infections in clinical settings^15^. Sulfonamides could compete with p-amino benzoic acid (PABA) for inclusion since they are structurally similar to it and may be needed by bacteria to synthesis vitamin Bc^8^. Infections in cattle herds are treated with sulfonamide antibiotics in veterinary medicine^16,17^. Bacterial resistance and sulfonamide adverse effects are two factors that limit the use of sulfonamides in therapy in order to overcome the challenge and climb continuous attempts are undertaken to create novel antibacterial compounds with the sulfonamide structure and to create novel formulations using the currently used sulfonamide substances in order to counteract the negative effects^18–20^. Due to the presence of sulfonamides-resistant dihydropteroate synthetic enzymes in these *E. coli* strains, resistance to sulfonamides has been demonstrated^21–23^. Throughout this research, new sulfonamide hybrids were synthesized and their anti-bacterial efficiency was determined using IZD and MIC techniques across both Gram+ and − strains. The synthesized hybrids were also subjected to modelling and docking studies.

## Results and discussion

### Synthesis

**Figure 2:**
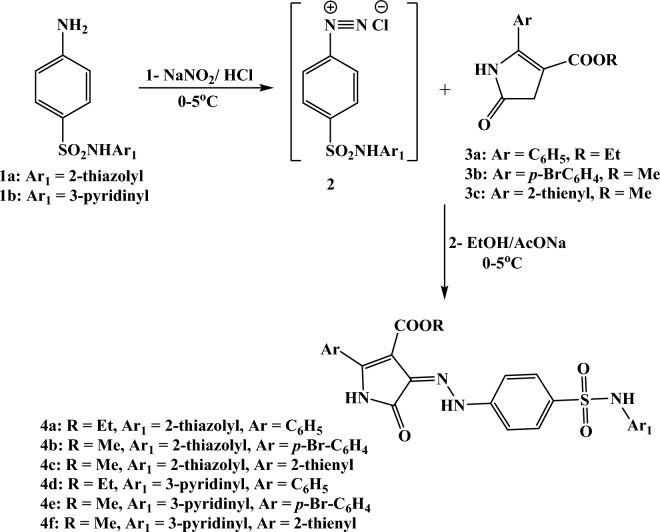
Synthesis of the new sulfonamide dyes **4a-f** (Created by: ChemDraw (Ver. 17)).

Two 4-amino-*N*-(aryl)benzenesulfonamide derivatives **1a** and** 1b** were diazotized upon conduct with (HCl/NaNO_2_) at 0–5 °C and the corresponding diazonium salts were coupled with three types of alkyl 2-aryl-4,5-dihydro-5-oxo-1*H*-pyrrole-3-carboxylate hybrids **3a**, **3b** or **3c**^24–26^ The diazotization response was effectively continued in EtOH solution and CH_3_COONa at 0–5 °C to furnish the targeting 5-oxo-2-phenyl-4-(arylsulfamoyl)phenyl)hydrazono)-4,5-dihydro-1*H*-pyrrole-3-carboxylate dyes **4a-f**. The proposed hybrids **4a-f** were available based on the compatible spectral information (Fig. 2).

**Figure 3:**
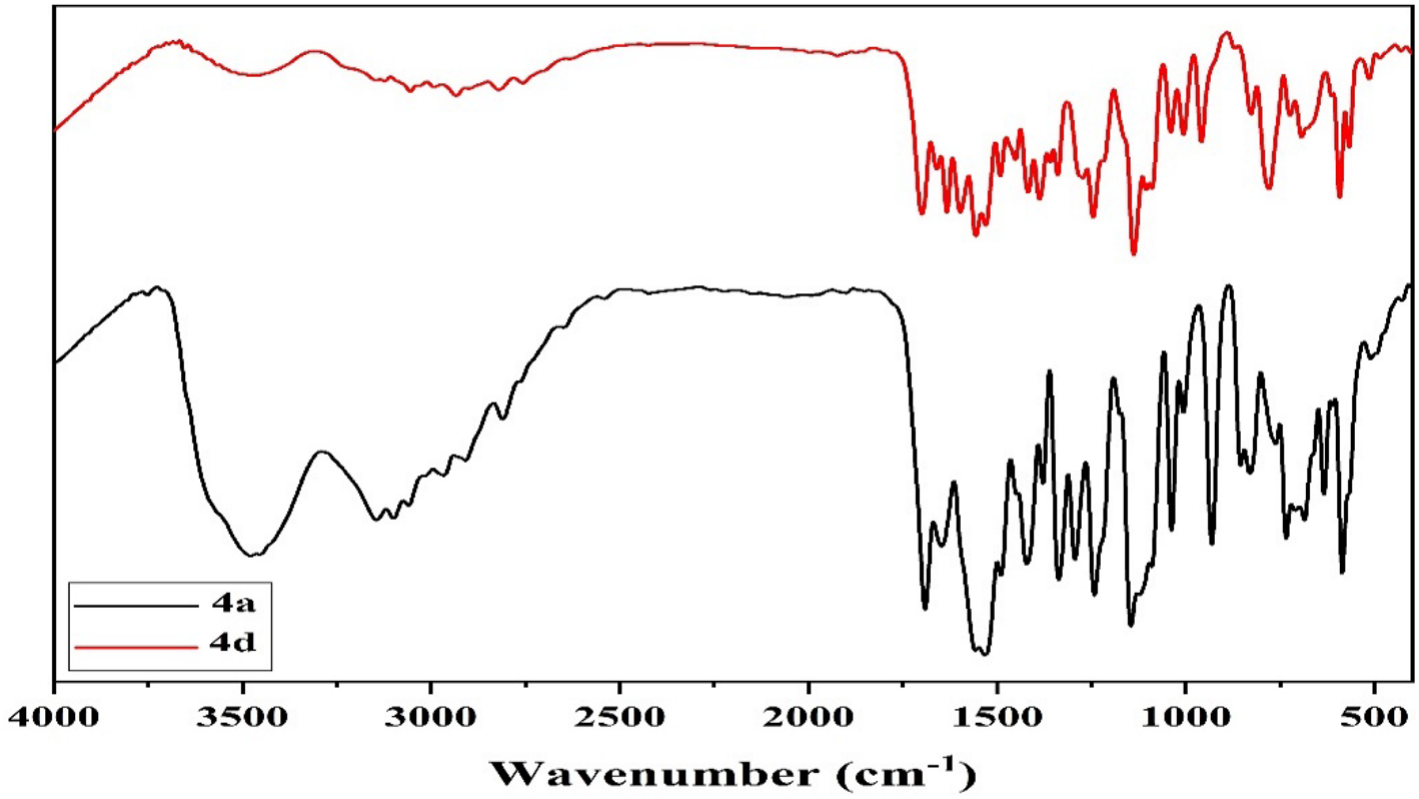
IR spectra of dyes **4a** and **4d** (Created by: OriginPro 2018 (Ver. SR1)).

A careful inspection of the synthesized derivatives IR spectra indicated that all derivatives are existing in keto-hydrazo (Fig. 3 and Figure S1) form where:

(i) The strong band at 3471–3425 cm^−1^ with a shoulder at 3265–3200 cm^−1^ were attributed to the stretching vibrations of the free and H-bonded pyrrole υ(NH)^27^, respectively. The band observed at 1668–1650 cm^−1^ region was assigned to the ν(C=O) vibration of oxo-pyrrole^27,28^. The spectra displayed another strong band at 1725–1690 cm^−1^ corresponding to the ν(C=O) of carboxylate group^27^.
(ii) Furthermore, the two bands at 3200–3140 and 3130–3100 cm^−1^ were assigned to the ν(NH) vibrations of sulfonamide^29^ and hydrazo^28^ groups, respectively. While, the sulfonamide υ(SO_2_)_s_ and υ(SO_2_)_as_ were displayed at 1374–1357 and 1144–1135 cm^−128^, respectively.
(iii) Moreover, the spectral data showed two bands only in the 1640–1630 and 1600–1590 cm^−1^ regions where the former was attributed to the overlapped hydrazo and heterocyclic ring υ(C=N) vibration^28^ while the latter was corresponding to the υ(C=C) of the aromatic rings^28^, respectively.
(iv) Also, the spectra displayed several bands at 1560–1555, 1250–1240, 1165–1145 and 735–680 cm^−1^ owing to the Amide II, Amide III, υ(N=NH) and ρ(NH) vibration^27–29^, respectively.

The ^1^H NMR spectrum of compound 4a (as an example) disclosed triplet (t) and quartet (q) signals (s) at δ 1.15 and 4.12 ppm belongs the protons of ethoxy group (-OCH_2_CH_3_). The protons of thiazole ring were detected as two doublet signals at δ 6.78 and 7.19 ppm. The aromatic protons were observed as multiplet and doublet signals in the region from δ 7.45 to 7.74 ppm. Meanwhile, protons of imino-groups were resonated as singlet signals at δ 11.40, 12.66, and 12.99 ppm.

### DFT calculation

**Figure 4:**
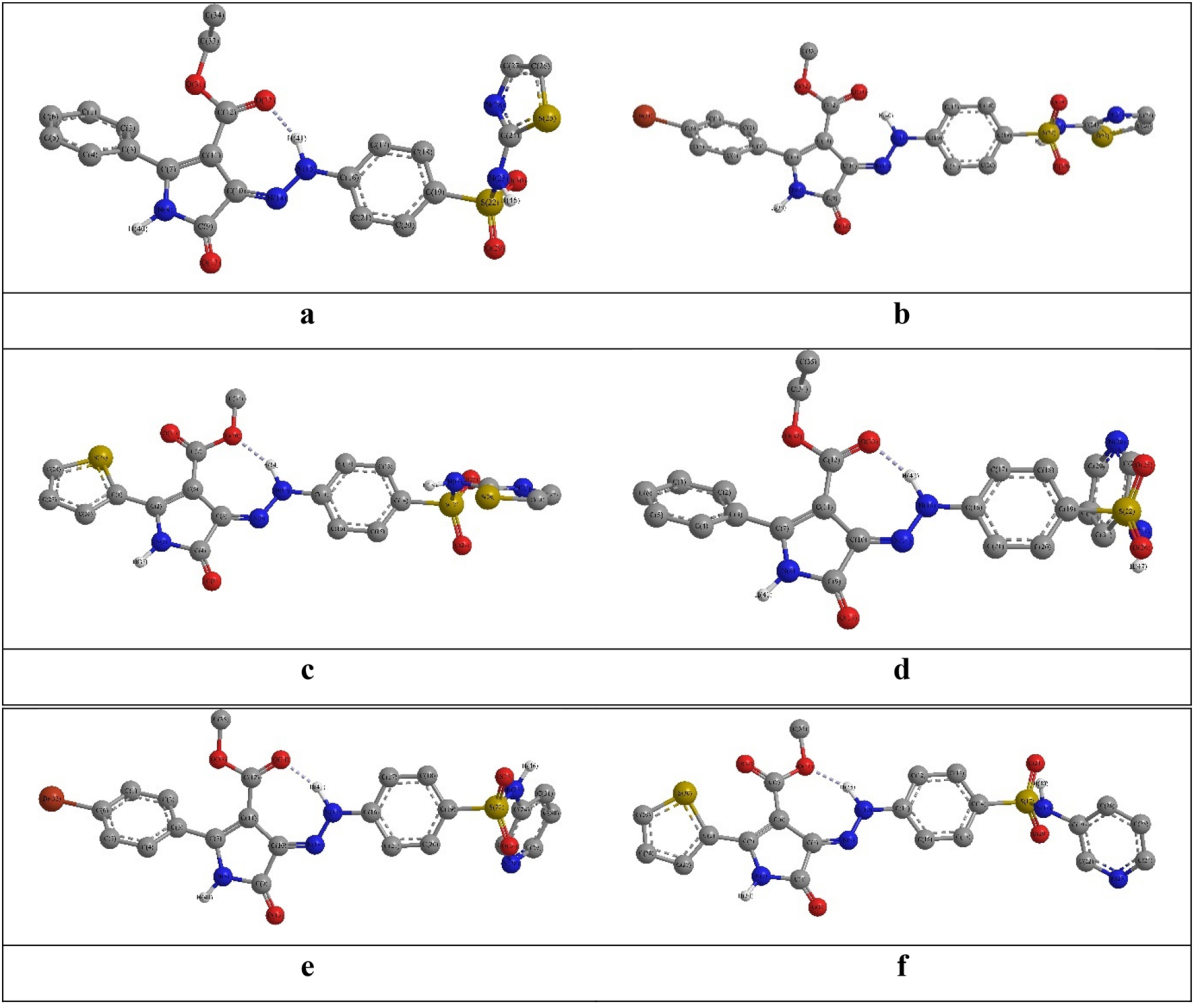
The DFT Optimized structures of the investigated dyes (Created by: GaussView (Ver. 6)).

The DFT geometrical optimization procedures presented resemble angular configuration of the investigated compounds in which the phenylhydrazineylidene pyrrole-3-carboxylate moiety has planar structure (Fig. 4). Whereas, the phenyl pyrrole substituent was tilted one the pyrrole ring plane, i.e., the C^2^_(PhPr)_–C^1^_(PhPr)_–C^2^_(Pr)_–N^1^_(Pr)_ =  − 133.2–135.8° and C^2^_(PhPr)_–C^1^_(PhPr)_–C^2^_(Pr)_–C^3^_(Pr)_ = 44.5–48.3°, Likewise, the thiophene ring in **4c** and **4f** derivatives was slanted on the pyrrole plane by 18.0° where the dihedral angle S^1^_(Tph)_–C^2^_(Tph)_–C^2^_(Pr)_–N^1^_(Pr)_ = 164.8° and S^1^_(Tph)_–C^2^_(Tph)_–C^2^_(Pr)_–C^3^_(Pr)_ = 18.2°. On the other hand, although the sulfonamide sulfur atom was coplanar with the phenyl ring, the nitrogen and oxygen atoms were strongly shifted out the phenyl ring plane, i.e., the C^3^_(Ph)_–C^4^_(Ph)_–S_(Sul)_–NH_(Sul)_ = 68.4–87.9° and C^3^_(Ph)_–C^4^_(Ph)_–S_(Sul)_–O _(Sul)_ = 161.6–178.2°. Consequently, the thiazole and pyridine rings were tilted on the sulfonamide S–NH as shown in the dihedral angles S_(Sul)_–NH_(Sul)_–C^2^_(Thz)_–N^3^_(Thz)_ = 29.0–42.2° and S_(Sul)_–NH_(Sul)_–C^3^_(Py)_–C^2^_(Py)_ = 54.7–80.7°, respectively (Table S1).

Moreover, both of bond length and angle data displayed noteworthy resemblance with those obtained from the X-ray of hybrid–single crystal^30,31^, where the lengths were longer than the corresponding X-ray by maximum 0.12 Å, RMSD 0.04–0.05, however the angles differences were in range 0.0–18.5°, RMSD = 5.6–6.7. These discrepancies may be attributed to that no intermolecular columbic interactions were considered in the DFT calculations as it carried out for a single molecule in gaseous state, while in practical, there is quite a few interrelating molecules in solid crystal lattice^32^ (Tables S2–S3).

**Figure 5:**
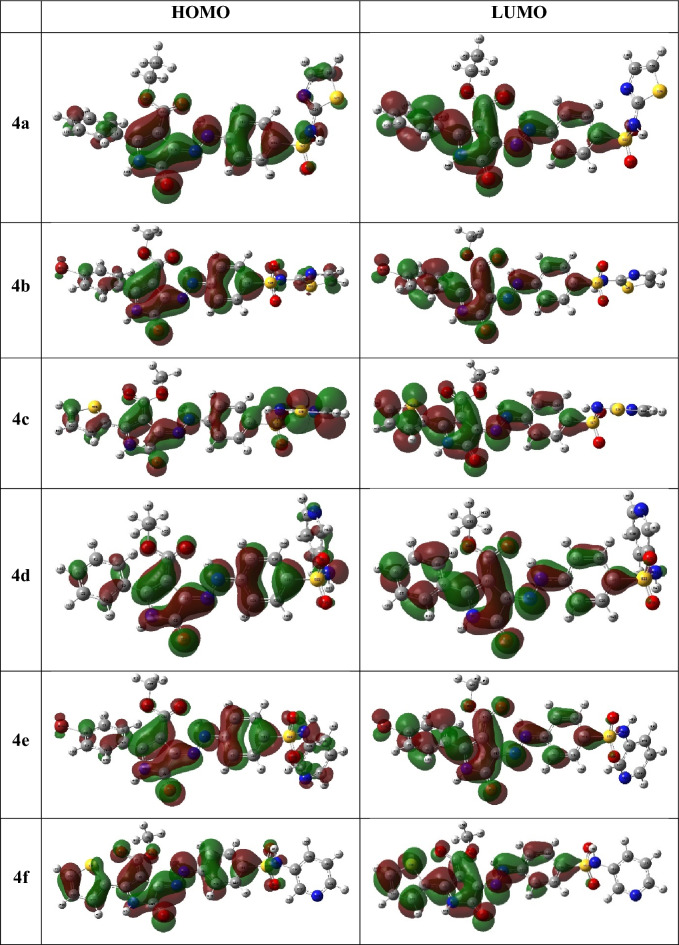
The frontier molecular orbital of the synthesized compounds **4a-f** (Created by: GaussView (Ver. 6)).

Furthermore, the HOMO and LUMO, frontier orbitals, using GaussView, version 5; 2009 program, have substantial role in molecule’s affinity to donate and accept electrons^33^, correspondingly, lengthways with molecule’s bioactivity that essentially affected by the HOMO–LUMO energy gap^34–36^. The graphs of frontier orbitals of deliberate hybrids displayed similar HOMO configuration which was primarily built up of the π-orbitals of conjugated system of the whole molecule as well as lone pairs of heteroatoms. Whereas, their LUMO were constructed predominantly from the π*-orbitals of the 5-oxo-2-phenyl-4-(arylsulfamoyl)phenyl)hydrazono)- pyrrole-3-carboxylate moiety (Fig. 5). Accordingly, alike configuration of the HOMO and LUMO led to close values of their energies where the E_H_ ranged from − 5.74 to − 6.00 eV, while the E_L_ were − 4.01 to − 4.28 eV and exhibited the same order, **4a** < **4d** < **4e** < **4f** < **4b** < **4c**. Also, the investigated hybrids revealed low and close energy gap (ΔE_H-L_), 1.68–1.75 eV, and may be sorted as **4b** < **4e** < **4c** < **4a** < **4f** < **4d** (Table 1).

**Table 1:** The HOMO–LUMO energies and chemical reactivity descriptors (eV) of investigated compounds.

Compound	E_H_	E_L_	ΔE_H-L_	χ	η	δ	ω	ω^+^	ω^−^
**4a**	− 5.74	− 4.01	1.73	4.88	0.87	1.15	13.72	11.39	16.26
**4b**	− 5.95	− 4.27	1.68	5.11	0.84	1.19	15.50	13.05	18.16
**4c**	− 6.00	− 4.28	1.72	5.14	0.86	1.16	15.39	12.92	18.06
**4d**	− 5.87	− 4.12	1.75	4.99	0.87	1.15	14.29	11.90	16.89
**4e**	− 5.93	− 4.23	1.70	5.08	0.85	1.17	15.17	12.73	17.81
**4f**	− 5.95	− 4.22	1.73	5.08	0.87	1.15	14.91	12.48	17.56

Moreover, chemical reactivity descriptors, like electronegativity (χ), global hardness (*η*), softness (*δ*), electrophilicity (*ω*), electron-donating power (*ω*^−^) and electron-accepting power (*ω*^+^) were computed using the E_H_ and E_L_ as follows^34^ (Eqs. 1–6).

1 $$\chi =-\frac{1}{2}\left({E}_{HOMO}+{E}_{LUMO}\right)$$

2 $$\eta =-\frac{1}{2}\left({E}_{HOMO}-{E}_{LUMO}\right)$$

3 $$\delta =\frac{1}{\eta }$$

4 $$\omega =\frac{{\chi }^{2}}{8\eta }$$

5 $${\omega }^{-}=\frac{{\left(3I+A\right)}^{2}}{16(I-A)}$$

6 $${\omega }^{+}=\frac{{\left(I+3A\right)}^{2}}{16(I-A)}$$

As shown in Table 1, the synthesized sulfonamide-based pyrrole compounds unveiled the lowest global hardness (η) and highest softness (δ) values and thus it is the furthermost reactive, slightest stable kinetically and softest one. Furthermore, the studied hybrids are powerful electrophile, ω = 13.72–15.50 eV, as strong electrophile has electrophilicity index ω > 1.5 eV^37,38^, and follow the order **4a** < **4d** < **4f** < **4e** < **4c** < **4b**. As well, the electron donating (ω^+^) and acceptance (ω^−^) powers of the inspected hybrids, which established the capability to donate and accept electrons, individually, conformed the impervious alignment nonetheless they revealed additional donation current, 11.39–13.05 eV, than acceptance, 16.26–18.16 eV, wherever slighter values indicate improved transaction^37,38^ (Table 1).

The Mulliken’s atomic charges offered interpretation of charge transfer and electronegativity of the molecule^39^. The atomic charges of the investigated hybrids showed that the pyrrole nitrogen atom, N^1^_(Pr)_, has a negative charge, − 0.488–0.495, while the C^5^_(Pr)_ atom has positive charge, 0.210–0.216, which may be attributed to the electron withdrawing effect of the nitrogen and attached oxygen atoms. Although the carbon atoms C^3^_(Pr)_ and C^4^_(Pr)_ have negative charge but the former was more negatively charge than the latter, − 0.125–0.152 and − 0.078–0.087, respectively, which may be originated from the difference between the carboxylate and phenyl hydrazinylidene groups electron affinity (Table 2). Moreover, the nitrogen and oxygen atoms acquired negative charge whereas the sulfur atoms of the sulfonamide group in addition to those of the thiazole and thiophene rings have positive charge which may be attributed to the presence of adjacent strong electron withdrawing atoms or their involvement in resonance structure of the heterocycle (Table 2).

**Table 2:** The Mulliken’s atomic charges (a.u.) of investigated compounds.

Atom	4a	4b	4c	4d	4e	4f
N^1^_(Pr)_	− 0.489	− 0.488	− 0.495	− 0.490	− 0.491	− 0.495
C^2^_(Pr)_	0.273	0.270	0.397	0.274	0.278	0.396
C^3^_(Pr)_	− 0.161	− 0.152	− 0.125	− 0.160	− 0.147	− 0.125
C^4^_(Pr)_	− 0.087	− 0.078	− 0.083	− 0.087	− 0.085	− 0.084
C^5^_(Pr)_	0.211	0.216	0.211	0.212	0.215	0.210
O_(OxPr)_	− 0.305	− 0.302	− 0.301	− 0.304	− 0.299	− 0.302
CO_(car)_	0.438	0.414	0.411	0.438	0.406	0.409
O^1^C_(car)_	− 0.442	− 0.439	− 0.373	− 0.443	− 0.430	− 0.374
O^2^C_(car)_	− 0.262	− 0.252	− 0.342	− 0.262	− 0.255	− 0.340
N^40^	− 0.079	− 0.073	− 0.081	− 0.081	− 0.078	− 0.081
NH^40^	− 0.386	− 0.381	− 0.377	− 0.384	− 0.386	− 0.376
C^1^_(Ph)_	0.465	0.439	0.468	0.459	0.466	0.472
C^4^_(Ph)_	− 0.090	− 0.189	− 0.194	− 0.094	− 0.087	− 0.093
S_(Sul)_	0.108	0.146	0.159	0.115	0.111	0.112
O^1^_(Sul)_	− 0.535	− 0.500	− 0.492	− 0.521	− 0.527	− 0.516
O^2^_(Sul)_	− 0.472	− 0.469	− 0.479	− 0.516	− 0.496	− 0.508
NH_(Sul)_	− 0.700	− 0.683	− 0.682	− 0.715	− 0.756	− 0.746
C^1^_(PhPr)_	0.246	0.251		0.247	0.257	
C^4^_(PhPr)_	− 0.252	0.195		− 0.251	0.196	
S^1^_(Thz)_	0.227	0.205	0.214			
C^2^_(Thz)_	0.167	0.136	0.124			
N^3^_(Thz)_	− 0.196	− 0.150	− 0.147			
C^4^_(Thz)_	− 0.178	− 0.184	− 0.182			
C^5^_(Thz)_	− 0.456	− 0.455	− 0.454			
N^1^_(Py)_				− 0.126	− 0.141	− 0.138
C^2^_(Py)_				− 0.334	− 0.260	− 0.263
C^3^_(Py)_				0.395	0.452	0.415
C^4^_(Py)_				− 0.320	− 0.338	− 0.328
C^5^_(Py)_				− 0.194	− 0.189	− 0.201
C^6^_(Py)_				− 0.271	− 0.284	− 0.264
S^1^_(Tph)_			0.386			0.384
C^2^_(Tph)_			− 0.154			− 0.153
C^3^_(Tph)_			− 0.297			− 0.298
C^4^_(Tph)_			− 0.249			− 0.249
C^5^_(Tph)_			− 0.469			− 0.469
Br		− 0.107			− 0.108	

Furthermore, the molecular parameters such as polarizability (α_total_), hyperpolarizabilities (β_total_), and dipole moment (μ), were calculated^40–42^ as shown below, as a measure for the molecule’s softness and electron density distribution that mainly influence the intermolecular interactions^43^, as well as, optical nonlinearity and response^44–47^.

7 $$\upmu =({\upmu }_{{\text{x}}}^{2}+{\upmu }_{{\text{y}}}^{2}+{\upmu }_{{\text{z}}}^{2})$$

8 $${\mathrm{\alpha }}_{{\text{total}}}=\frac{({\mathrm{\alpha }}_{{\text{xx}}}+{\mathrm{\alpha }}_{{\text{yy}}}+{\mathrm{\alpha }}_{{\text{zz}}})}{3}$$

9 $${\upbeta }_{{\text{total}}}=\sqrt{{\left({\upbeta }_{{\text{xxx}}}+{\upbeta }_{{\text{xyy}}}+{\upbeta }_{{\text{xzz}}}\right)}^{2}+{\left({\upbeta }_{{\text{yyy}}}+{\upbeta }_{{\text{yzz}}}+{\upbeta }_{{\text{yxx}}}\right)}^{2}+{\left({\upbeta }_{{\text{zzz}}}+{\upbeta }_{{\text{zxx}}}+{\upbeta }_{{\text{zyy}}}\right)}^{2}}$$

The explored hybrids dipole moments (μ) were ranged from 10.39 D, for hybrid **4f**, to 12.8 D, for hybrid **4d** subsequent the order **4f** < **4c** < **4e** < **4a** < **4b** < **4d** (Table 3). Meanwhile, the polarizability (α_total_) data of the inspected hybrid demonstrated nearby values, wherever the hybrids **4a** and **4b** unveiled the lowest and highest values, 2.87 × 10^–23^ and 3.34 × 10^–23^ esu, correspondingly. However, the 1st order hyperpolarizability facts of the explored hybrids discovered that the bigger value was detected for the hybrid **4d**, 7.70 × 10^–30^ esu, whereas **4a** has the lowest, β_total_ = 4.89 × 10^–30^ esu. On judgement with the matching value documented for the urea reference^48^, it was noticed that all explored hybrids have match more hyperpolarizability over urea by lower 13.09 to maximum 20.59 times and may be arranged as **4a** < **4c** < **4f** < **4e** < **4b** < **4d**, correspondingly (Table 3).

**Table 3:** The calculated dipole moment (μ), polarizability (α_total_), polarizability anisotropy (Δα) and first-order hyperpolarizability (β_total_) of examined compounds.

Compound	μ (Debye)	μ/μ_urea_	α_total_ (esu × 10^–23^)	Δα (esu × 10^–24^)	β_total_ (esu × 10^–30^)	β_total_/β_urea_
**4a**	10.88	7.92	2.87	8.18	4.89	13.09
**4b**	12.02	8.75	3.34	9.16	7.35	19.65
**4c**	10.65	7.76	2.92	6.45	5.34	14.27
**4d**	12.89	9.39	3.07	7.63	7.70	20.59
**4e**	10.86	7.91	3.26	9.96	7.19	19.22
**4f**	10.39	7.57	2.91	8.88	5.91	15.79

### In vitro antibacterial activity

**Figure 6:**
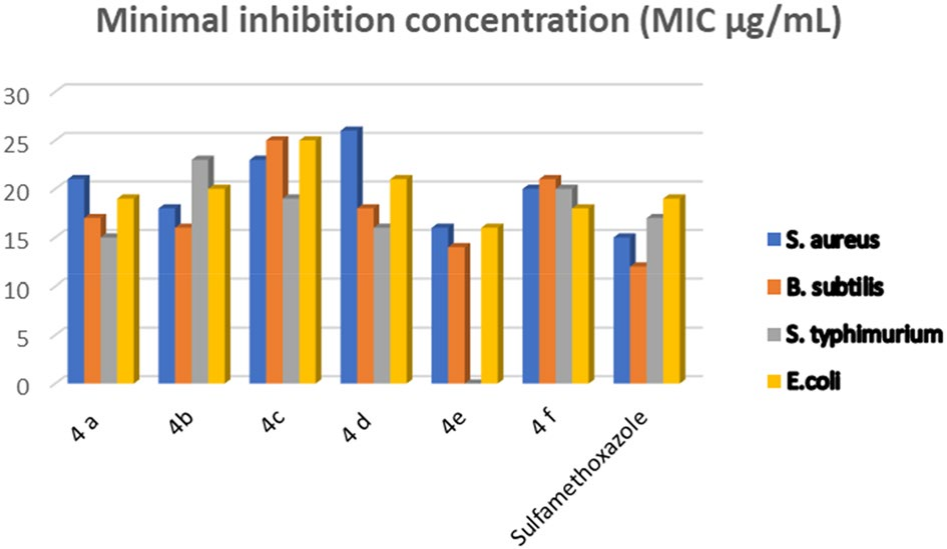
MIC of the explored hybrids over both of Gram + and Gram—bacteria.

In accordance to, both of IZD and MIC methodology, the synthesized sulfonamide hybrids were inspected over antibacterial efficacies crossways Gram+ and − strains. Both of table S4 and Fig. 6 were recognized the antimicrobial actions of the synthesized hybrids over the antibacterial effectiveness since sulfonamides have the competence to stop the generation of folic acid in the bacterial growing^49^. Through the explored hybrids, they unveiled more liable outcomes towards both of “*S. aureus* and *B. subtilis”* Gram + and “*S. typhimurium* and *E. coli”* Gram -ve bacterial straining cells. Where, sulfonamide hybrids **a-f** designated respectable activity in general, particularly through Gram + bacteria higher than Gram—proportional to Sulfamethoxazole reference. Temporarily, sulfonamide hybrid 4**a** have both of amino moiety and thiazole ring exhibited good inhibition zone of (IZD = 17 mm, MIC = 11.31 µg/mL) towards *B. subtilis*, near to the Sulfamethoxazole (IZD = 16 mm, MIC = 11.31 µg/mL), and weak inhibition zone of (IZD = 21 mm, MIC = 11.31 µg/mL) towards *S. aureus*. However, sulfonamide derivative **4b** have a pyridine ring revealed amazing inhibition (IZD = 16 mm, MIC = 11.33 µg/mL) against *B. subtilis* and better inhibition to *S. aureus* with (IZD = 18 mm, MIC = 11.33 µg/mL) with a match to reference (IZD = 17 mm, MIC = 11.31 µg/mL). Though, sulfonamide derivative **4c** exhibited a poor inhibition to the Sulfamethoxazole reference towards *S. aureus* with (IZD = 23 mm, MIC = 11.31 µg/mL) and weak inhibition over* B. subtilis* with (IZD = 25 mm, MIC = 11.31 µg/mL). Also, sulfonamide derivative **4d** showed the weakest inhibition against *S. aureus* with (IZD = 26 mm, MIC = 11.31 µg/mL) and better inhibition over *B. subtilis* with (IZD = 18 mm, MIC = 11.31 µg/mL). Whereas, sulfonamide derivative **4e** have displayed potent inhibitions over both of *S. aureus* and *B. subtilis* with (IZD = 16, 14 mm, MIC = 19.24, 11.31 µg/mL), respectively. Although, sulfonamide derivative **4f** presented a weak inhibition to the Sulfamethoxazole reference towards *S. aureus* and *B. subtilis* with (IZD = 20 mm, MIC = 11.31 µg/mL) and (IZD = 21 mm, MIC = 11.31 µg/mL), respectively.

Also, the results of hybrids **a-f** against Gram−bacteria “*S. typhimurium* and *E. coli*” displayed a proper reactivates. Where, hybrid **4a** presented potent inhibition over *S. typhimurium* through (IZD = 15 mm, MIC = 19.24 µg/mL) and a significant inhibition with (IZD = 19 mm, MIC = 11.31 µg/mL) over *E. coli* in contrast to Sulfamethoxazole (IZD = 19, 20 mm, MIC = 11.31 µg/mL), respectively*.* While, hybrid **4b** revealed weak inhibition over* S. typhimurium* with (IZD = 23 mm, MIC = 11.31 µg/mL) and a proper inhibition with (IZD = 20 mm, MIC = 11.31 µg/mL) over* E. coli*. Moreover, hybrid **4c** revealed potent inhibition towards* S. typhimurium* with (IZD = 19 mm, MIC = 19.24 µg/mL) and very poor inhibition with (IZD = 25 mm, MIC = 11.31 µg/mL) against* E. coli*. Whereas, hybrid **4d** demonstrated potent inhibition with (IZD = 16 mm, MIC = 19.24 µg/mL) over *S. typhimurium* with weak inhibition over *E. coli* with (IZD = 21 mm, MIC = 11.31 µg/mL). Meanwhile, hybrid **4e** wasn’t display any inhibition 19.24 but it showed an eminent inhibition towards *E. coli* with (IZD = 16 mm, MIC = 19.24 µg/mL) against *E. coli*. Furthermore, hybrid **4f** publicized superior inhibition against* S. typhimurium* with (IZD = 20 mm, MIC = 19.24 µg/mL) and an excellent inhibition with (IZD = 18 mm, MIC = 11.31 µg/mL) against* E. coli* (Table S4).

### Structural activity relationship

Because all of the synthesized hybrids contain a sulfonamide moiety, however several compound contain sulphonamide bond play an important role in enhancing the bioactivity^50^, the research was motivated by the intriguing reactivity results of sulfonamide hybrids **a–f** towards various bacterial strains^5^. The thiazole ring in sulfonamide derivatives plays a crucial role in bacterial death by preventing the manufacture of folic acid, and the hybrids **4a**, **4d**, and **4e** that include it were shown to have acceptable activations against the two bacterial strains as a result^51^, a substance that is necessary for bacterial development and reproduction. Sulfonamides successfully prevent bacterial reproduction by interfering with this metabolic pathway, enabling the immune system to eradicate the infection^52^. Additionally, sulfonamides with pyridine moiety, such as those found in hybrids **4b**, **4e**, and **4f**, work to inhibit the enzyme use PABA, a crucial metabolite for bacterial growth, in order to exhibit their antibacterial actions^53^ The pyridine ring also improves these antibiotics' solubility and cellular penetration, which adds to their total antibacterial efficacy. Sulfonamides with a pyridine ring efficiently limit bacterial proliferation and help treat bacterial infections by targeting important metabolic processes and bacterial enzymes^54,55^.

### Molecular docking

**Figure 7:**
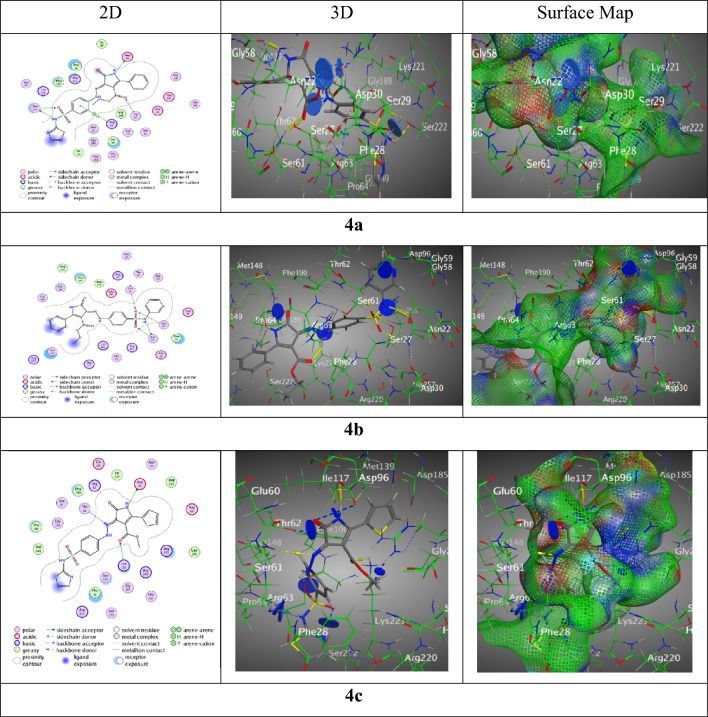
Interaction bindings of hybrids **4a-c** and 3TZF (Created by: MOE (Ver. 2019)).

Tables S5 and S6 provide the fallouts of molecular docking performed on compounds **4a-f** by M.O.E “v10.2019.01” computer software. The purpose of this was to develop hypotheses about the binding of the most active compounds to the PABA constituent of *S. aureus* strain. To create the model for this site, the researchers used the X-ray crystal structures of both a wild type enzyme DHPS (Dihydroptorate Synthase of Versinia pestis, PDB: ID 3TZF)^56^ and *S. aureus* F17L/E208K double mutant DHPS in the ligand-bound conformation with the PDB ID of 6CLV. The study focused on examining sulfonamide-based analogues and their bindings with the amino acids in the 6CLV structure^51^. In the interactions with (BDB:3TZF), hybrid **4a** exhibited a binding affinity of − 7.78 kcal/mol, with interactions spanning hydrogen bonds and π-H interactions across four different residues (Asp96, Ser222, Thr62, and Arg63), indicating a diverse and strong interaction profile, contributing to its high binding affinity. The RMSD value of 1.56 indicates a stable docking posture with distances ranging from 2.68 to 4.37 Å, emphasizing the significance of spatial location for efficient binding. Similarly, hybrid **4b** exhibited a binding affinity of − 7.70 kcal/mol, mostly participating in hydrogen acceptor interactions, demonstrating the compound's potential to create stable hydrogen bonds with the targeted protein. Its interactions are defined by distances that permit efficient binding, demonstrating a strong interaction despite having a somewhat lower binding affinity than **4a**. Hybrid **4c**, with the greatest binding affinity of − 8.09 kcal/mol in the series, had a high tendency for hydrogen bonding, suggesting a very favorable interaction with the protein target. The low RMSD value of 1.28 adds to the dependability of its binding position, making it the most viable choice for future exploration. (Fig. 7).

However, Hybrid **4d** exhibited a binding affinity of − 7.54 kcal/mol with a combination of hydrogen donor and acceptor interactions, indicating flexible binding capabilities. The contact distances and diversity of bonds indicate a balanced interaction profile, which, despite its lower affinity, highlights the compound's potential for specificity.

**Figure 8:**
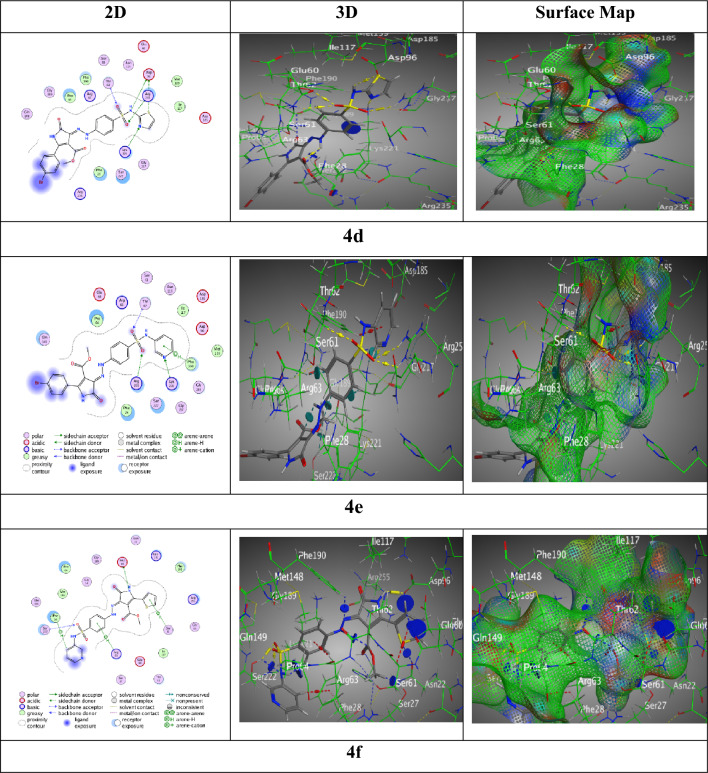
Interaction bindings of hybrids** 4d-f** and 3TZF (Created by: MOE (Ver. 2019)).

Meanwhile, hybrid **4e** demonstrated a binding affinity of -7.61 kcal/mol and participated in hydrogen acceptor and π-H interactions, indicating a high capacity to interact with critical residues. The comparatively low RMSD value of 1.42 demonstrates high confidence in the docking data, suggesting that **4e**'s interaction profile is favorable for successful binding. Hybrid **4f** showed a binding affinity of -7.68 kcal/mol and a complicated interaction pattern with hydrogen bonding and π interactions, indicating a multidimensional approach to binding. The low RMSD value of 1.13 indicates a very precise docking posture, indicating a considerable potential as a protein inhibitor (Fig. 8).

**Figure 9:**
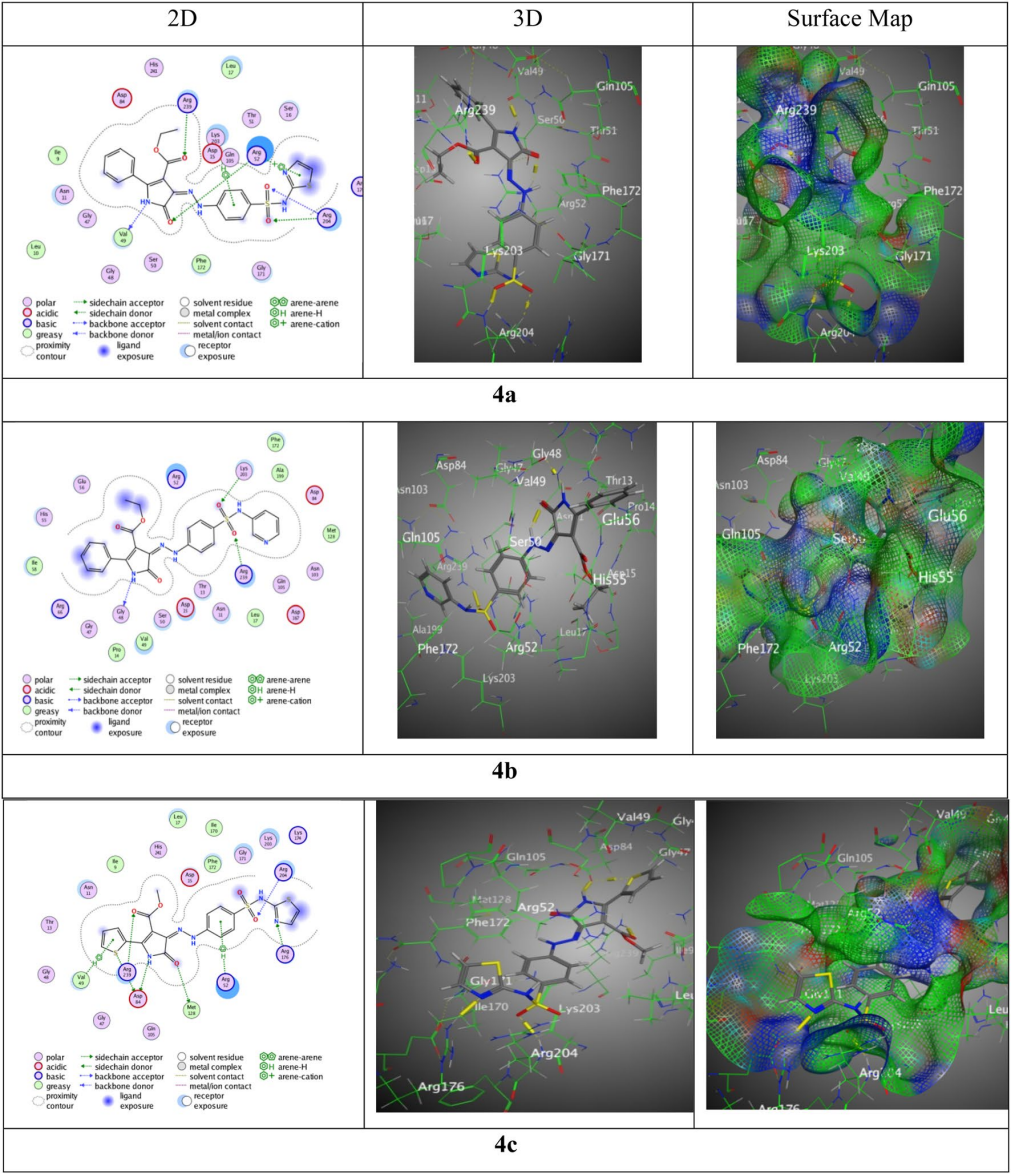
Interaction bindings of hybrids **4a-c** and 6CLV (Created by: MOE (Ver. 2019)).

Wherever, the interactions with (BDB: 6CLV) hybrid **4a** was displayed good score of interaction S = − 7.29 kcal/mol along RMSD = 1.44, via one H-donor between N 8 of pyrazolone ring with Val49, three H-acceptors between O12 of pyrazolone, O 28 and O 29 with Arg204, O 31f. ester moiety with Arg239, thiazole ring with Arg52 over π-cation, and phenyl ring of sulfonamide group with Lys203 over π-H (Fig. 7). While, sulfonamide hybrid **4b** presented binding through N 8 of pyrazolone group with Gly48 over H-acceptor, O 24 and O 25 with Arg239 and Lys203, respectively over two H-acceptors resulted from a good score of interactions S = − 7.91 kcal/mol beside RMSD = 1.43. Nevertheless, a remarkable score of interactions for sulfonamide hybrid **4c** was observed by S = − 7.39 kcal/mol with RMSD = 1.41 through six H-bonds, three H-donors between N 3 of pyrazolone ring beside S28 of thiophene ring with Asp84, O 7 of pyrazole ring with Met128, three H-acceptors sideways between N 22 of thiazole ring with Arg176, O 23 of sulfonamide moiety with Arg204, O 30 of carbonyl ester with Arg239, and the phenyl ring of sulfonamide moiety with Arg52 through π-H interaction (Fig. 9).

**Figure 10:**
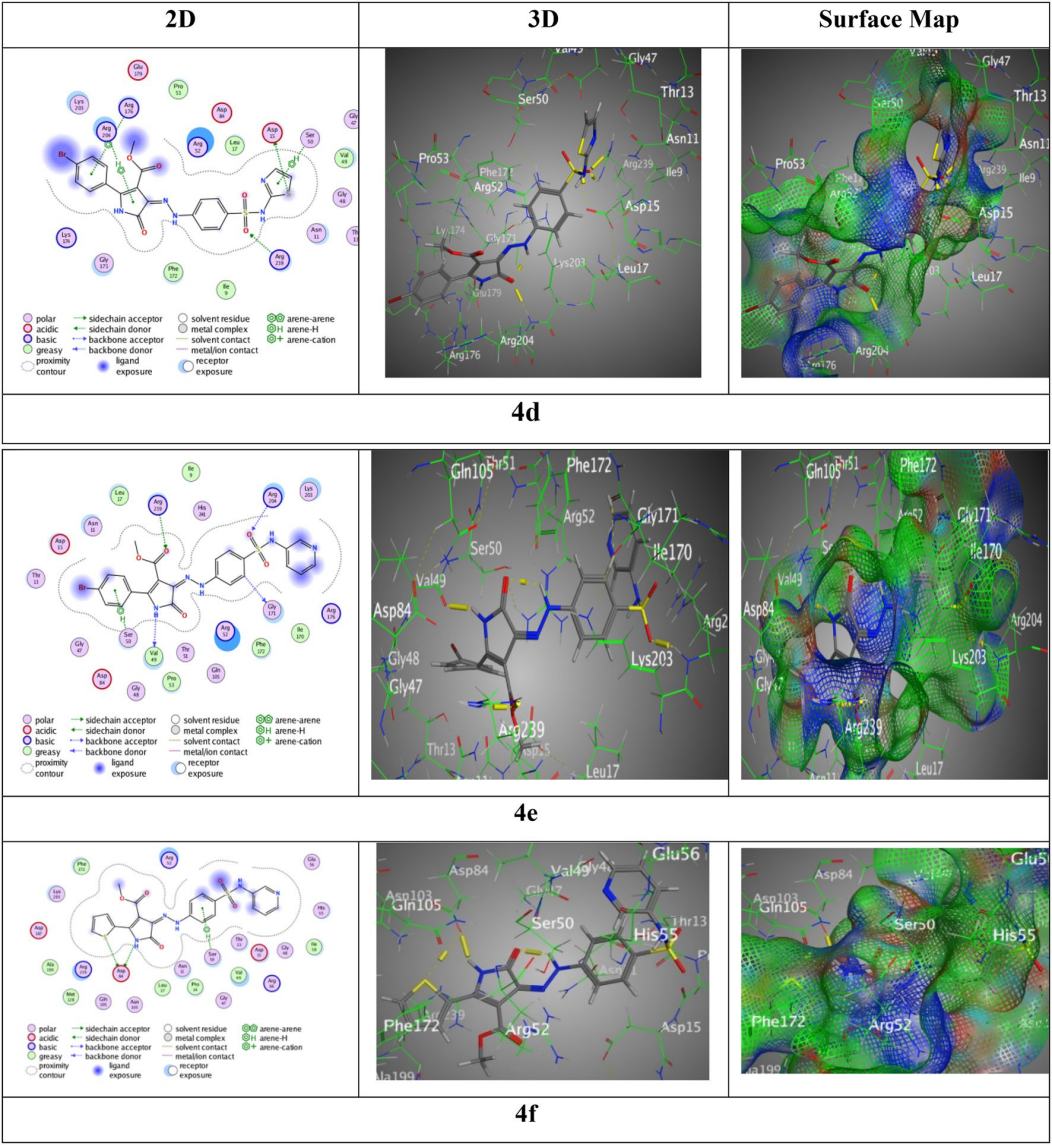
Interaction bindings of hybrids** 4d-f** and 6CLV (Created by: MOE (Ver. 2019)).

However, sulfonamide hybrid **4d** revealed score S = − 7.1042 kcal/mol along RMSD 1.4640 over S 24 of thiazole moiety with Asp15 through H-donor, O 29 of sulfonamide moiety with Arg239 by H-acceptor, thiazole ring with Ser50 through π-H, Arg204 with pyrazole through π-H, and Arg176 with bromo-phenyl ring through π-cation. Though, the highest score of interactions resulted in sulfonamide hybrid **4e** exhibited through S = − 8.0112 kcal/mol beside RMSD = 1.42 through four H-bonds, two H-donors among N 8 of pyrazole ring with Val49 and C 17 of phenyl of sulfonamide moiety with Gly171, two H-acceptors along O 25 with Arg204, O 33 with Arg239, and the bromo-phenyl ring with Ser 50 across π-H interaction. But, sulfonamide hybrid **4f** displayed score of interactions by S = − 7.7906 kcal/mol with adequate Rmsd = 0.96 through three interactions, two H-donors between Asp84 with N 3 of pyrazolone ring beside S 29 of thiophene ring, one π-H interaction amongst the phenyl ring of sulfonamide moiety with Ser50 (Fig. 10).

Through the binding energy, with hybrid **4e** exhibiting the most favourable binding energy of − 8.01 kcal/mol. Hybrid **4f**, in particular, has the lowest RMSD value of 0.96, indicating a solid and stable docking conformation. While all hybrids exhibit a combination of these interactions, hybrids **4a** and **4c** stand out for having a more diversified collection of interactions, including π-cation interactions.

The study on the impacts of docking revealed several findings: (1) the primary objective of docking simulation is to identify the most promising interaction between the chosen proteins and its potential partners, while considering other alternatives. (2) The comprehensive molecular docking analysis of the hybrid compounds **4a** through **4f** has elucidated their interaction mechanisms and binding affinities with a target (BDB:3TZF) protein, revealing significant insights into their potential as inhibitors. 3) Among the hybrids tested with the 6CLV protein, those containing pyrimidine substituents (hybrids **4e**, **4b**, and **4f**) had higher binding scores (− 8.01, − 7.91, and − 7.79 kcal/mol, correspondingly) related to the other hybrids. 3) Most of the derived compounds were connected to amino acids of 6CLV (Val49, Arg52, Arg204, Arg239, Asp84, and Ser50) through hydrogen bonds and π-H interactions. The analogues were represented in both two and three-images, as well as electron density maps.

### In silico ADME analysis

A complete investigation of physicochemical properties for many hybrid compounds was reported in Table S7 utilizing the Swiss ADME program. These characteristics are useful markers of a molecule's pharmacokinetics and drug-likeness. The effect of molecular weight on the ADME characteristics of compounds has been thoroughly studied. Orally active medicines typically have molecular weights ranging from 160 to 480 Da^57^. All of the hybrids described here meet these criteria. Meanwhile, molecular flexibility, as measured by the number of rotatable bonds, can have a considerable influence on molecule bioavailability. Compounds with fewer than ten rotatable bonds have higher oral bioavailability^58^. This requirement is met by all of the hybrids mentioned. Molar refractivity temporarily reveals molecule size and electrical properties. This characteristic has been linked to drug permeability^59^. TPSA also represents the molecule's capacity to interact with biological membranes. Increased TPSA usually indicates a problem with permeability^59^. The hybrid with the greatest TPSA among the hybrids may indicate a relative reduction in permeability. Also MLOGP, a representation of compound lipophilicity, is required because lipophilicity has a significant impact on the pharmacokinetic properties of compounds^60^. ESOL also provides predictions on the water solubility of compounds. All of the hybrids are labelled MS, which indicates moderate solubility, indicating that solubility may not offer substantial issues for these compounds^61^.

The Swiss ADME program was used to assess the physicochemical characteristics of the hybrids, which provided significant insights into their potential drug-like qualities. While certain hybrids have values that are outside of the commonly recognized range for oral medications, it is important to note that there are exceptions, and experimental evidence is required for conclusive findings. In general, these hybrids have an appealing profile, particularly in terms of hydrogen bonding and rotatable bonds. However, more *in-vivo* and *in-vitro* investigations would be required to determine their true pharmacokinetic and pharmacodynamic capabilities.

## Experimental

### Instruments

Melting points were recorded via Gallenkamp device. FT-IR spectra were recoded as KBr disc on FT-IR 6300 device, and υ_max_ was assigned in cm^−1^. ^1^H NMR and ^13^C NMR spectra were assessed with a Jeol device at 500 MHz for ^1^H NMR and 125 MHz for ^13^C NMR and DMSO-*d*_6_ as a solvent over TMS as standard. Chemical shifts are conveyed in δ. Mass spectrophotometry was evaluated via Thermoscientific EI (70 eV) manner. Perkin-Elmer 2400 analyzer has been utilized to assign the elemental analyses. Sulfathiazole (**1a**) and pyridine-3-sulfonamide (**1b**) are purchased from Sigma-Aldrich. Alkyl 2-aryl-4,5-dihydro-5-oxo-1*H*-pyrrole-3-carboxylate compounds **3a**, **3b** and **3c** were prepared previously according to the published methodology in the literature^24–26^.

## Chemistry

### Synthesis of 5-oxo-2-phenyl-4-(arylsulfamoyl)phenyl)hydrazono)-4,5-dihydro-1H-pyrrole-3-carboxylate compounds 4a-f

4-Amino-*N*-(aryl)benzenesulfonamide derivative **2** (0.01 mol) was dissolved in 40 mL H_2_O and concentrated HCl (35%, 3 mL), the combination was chilled down to 0–5 °C, and then diazotized with NaNO_2_ (0.69 g) in 10 mL H_2_O was added drop-wisely with stirring over 15 min over the suspended solution. The obtained diazonium solution was added to a suspension of each alkyl 2-aryl-4,5-dihydro-5-oxo-1*H*-pyrrole-3-carboxylate derivative** 1a**,** 1b** or** 1c** (1.00 mmol) in EtOH solution and CH_3_COONa at 0–5 °C. The coupling was stirred until pH was stabled. The precipitate arylazo-sulfonamide dyes **4a-f** was collected by filtration.

### Ethyl 5-oxo-2-phenyl-4-(2-(4-(N-(thiazol-2-yl)sulfamoyl)phenyl)hydrazono)-4,5-dihydro-1H-pyrrole-3-carboxylate (4a)

Red powder, recrystallized from EtOH, yield = 76%, m.p. = 199–201 °C. IR (KBr): 3471, 3223, 3145, 3100 (N–H), 1691, 1668 cm^−1^ (C=O), 1355, 1140 (SO_2_). ^1^H NMR: δ 1.15 (t, *J* = 6.70 Hz, 3H, -CH_3_), 4.12 (q, *J* = 6.70 Hz, 2H, –OCH_2_), 6.78 (d, *J* = 4.75 Hz, 1H, thiazole-H5), 7.19 (d, *J* = 4.75 Hz, 1H, thiazole-H4), 7.45–7.58 (m, 5H, Ar–H), 7.65 (d, *J* = 8.55 Hz, 2H, Ar–H), 7.74 (d, *J* = 8.55 Hz, 2H, Ar–H), 11.40 (s, 1H, N–H), 12.66 (s, 1H, N–H), 12.99 ppm (s, 1H, N–H). ^13^C NMR: δ 14.50, 60.22, 99.44, 108.58, 114.25 (2C), 128.14 (2C), 128.30, 128.55 (2C), 129.44 (2C), 145.99, 146.65, 148.79, 158.27, 161.77, 162.55, 164.78, 166.30, 169.18. Mass analysis (*m/z*, %): 497 (14.48%). Analysis for C_22_H_19_N_5_O_5_S_2_ (497.54): Calculated: C, 53.11; H, 3.85; N, 14.08%. Found: C, 53.26; H, 3.87; N, 14.13%.

### Methyl 2-(4-bromophenyl)-5-oxo-4-(2-(4-(N-(thiazol-2-yl)sulfamoyl)phenyl)hydrazono)-4,5-dihydro-1H-pyrrole-3-carboxylate (4b)

Red powder, recrystallized from EtOH, yield = 72%, m.p. = 234–236 °C. IR (KBr): 3450, 3200, 3200, 3112 (N–H), 1722, 1660 cm^−1^ (C=O), 1374, 1139 (SO_2_). ^1^H NMR: δ 3.66 (s, 3H, –OCH_3_), 6.76 (d, *J* = 4.70 Hz, 1H, thiazole-H5), 7.20 (d, *J* = 4.70 Hz, 1H, thiazole-H4), 7.48–7.52 (m, 4H, Ar–H), 7.64 (d, *J* = 8.55 Hz, 2H, Ar–H), 7.75 (d, *J* = 8.55 Hz, 2H, Ar–H), 11.46 (s, 1H, N–H), 12.64 (s, 1H, N–H), 13.02 ppm (s, 1H, N–H). ^13^C NMR: δ 51.65, 102.83, 108.59, 114.79 (2C), 124.40, 128.15 (2C), 128.37, 131.40 (2C), 131.60 (2C), 145.86, 147.79, 156.58, 161.66, 162.90, 164.72, 166.52, 169.18. Mass analysis (*m/z*, %): 563 (M^+^, Br-81, 22.16%), 561 (M^+^, Br-79, 22.73%). Analysis for C_21_H_16_BrN_5_O_5_S_2_ (562.41): Calculated: C, 44.85; H, 2.87; N, 12.45%. Found: C, 44.78; H, 2.91; N, 12.40%.

### Methyl 5-oxo-4-(2-(4-(N-(thiazol-2-yl)sulfamoyl)phenyl)hydrazono)-2-(thiophen-2-yl)-4,5-dihydro-1H-pyrrole-3-carboxylate (4c)

 Red solid, recrystallized from EtOH, yield = 73%, m.p. = 186–188 °C. IR (KBr): 3442, 3230, 3139, 3107 (N–H), 1692, 1661 cm^−1^ (C=O), 1361, 1144 (SO_2_). ^1^H NMR: δ 3.86 (s, 3H, –OCH_3_), 6.78 (d, *J* = 4.50 Hz, 1H, thiazole-H5), 7.19 (d, *J* = 4.50 Hz, 1H, thiazole-H4), 7.31–7.34 (m, 1H, thiophene-H), 7.68 (d, *J* = 8.50 Hz, 2H, Ar–H), 7.77 (d, *J* = 8.50 Hz, 2H, Ar–H), 7.88–7.90 (m, 2H, thiophene-H), 11.26 (s, 1H, N–H), 12.66 (s, 1H, N–H), 13.14 ppm (s, 1H, N–H). ^13^C NMR: δ 54.51, 107.37, 113.12, 119.24 (2C), 128.64, 129.85, 130.14, 131.83 (2C), 132.94, 136.05, 148.79, 158.87, 161.77, 162.55, 164.78, 166.30, 169.18. Mass analysis (*m/z*, %): 489 (27.17%). Analysis for C_19_H_15_N_5_O_5_S_3_ (489.54): Calculated: C, 46.62; H, 3.09; N, 14.31%. Found: C, C, 46.81; H, 3.02; N, 14.20%.

### Ethyl 5-oxo-2-phenyl-4-(2-(4-(N-(pyridin-3-yl)sulfamoyl)phenyl)hydrazono)-4,5-dihydro-1H-pyrrole-3-carboxylate (4d)

Orange powder, recrystallized from EtOH, yield = 83%, m.p. = 265–267 °C. IR (KBr): 3431, 3229, 3146, 3103 (N–H), 1699, 1654 cm^−1^ (C = O), 1368, 1130 (SO_2_). ^1^H NMR: δ 1.14 (t, *J* = 6.70 Hz, 3H, –CH_3_), 4.12 (q, *J* = 6.70 Hz, 2H, –OCH_2_), 7.12 (d, *J* = 4.70 Hz, 1H, pyridine-H), 7.45–7.57 (m, 5H, Ar–H), 7.64 (d, *J* = 8.55 Hz, 2H, Ar–H), 7.70–7.76 (m, 2H, pyridine-H), 7.82 (d, *J* = 8.55 Hz, 2H, Ar–H), 8.04 (s, 1H, pyridine-H), 11.41 (s, 1H, NH), 12.96 (s, 1H, NH), 13.06 ppm (s, 1H, NH). ^13^C NMR: δ 14.50, 60.22, 102.78, 114.00, 114.58 (2C), 128.30, 128.55 (2C), 128.85, 129.41 (2C), 130.88 (2C), 132.34, 135.71, 140.47, 146.23, 148.96, 153.39, 161.76, 158.37, 162.53, 166.13. Mass analysis (*m/z*, %): 491 (32.76%). Analysis for C_24_H_21_N_5_O_5_S (491.52): Calculated: C, 58.65; H, 4.31; N, 14.25%. Found: C, 58.78; H, 4.24; N, 14.18%.

### Methyl 2-(4-bromophenyl)-5-oxo-4-(2-(4-(N-(pyridin-3-yl)sulfamoyl)phenyl)hydrazono)-4,5-dihydro-1H-pyrrole-3-carboxylate (4e)

Reddish orange solid, recrystallized from EtOH, yield = 65%, m.p. = 154–156 °C. IR (KBr): 3460, 3265, 3194, 3130 (N–H), 1725, 1650 cm^−1^ (C=O), 1366, 1135 (SO_2_). ^1^H NMR: δ 3.61 (s, 3H, –OCH_3_), 7.13 (d, *J* = 4.50 Hz, 1H, pyridine-H), 7.48–7.53 (m, 4H, Ar–H), 7.62 (d, *J* = 8.00 Hz, 2H, Ar–H), 7.71–7.74 (m, 2H, pyridine-H), 7.85 (d, *J* = 8.00 Hz, 2H, Ar–H), 8.05 (s, 1H, pyridine-H), 11.35 (s, 1H, N–H), 12.87 (s, 1H, N–H), 13.24 ppm (s, 1H, N–H). ^13^C NMR: δ 53.23, 101.14, 112.35 (2C), 118.62 (2C), 122.31, 127.44 (2C), 129.13, 132.61 (2C), 133.29 (2C), 136.02 (2C), 139.50, 147.39 (2C), 151.67, 159.17, 166.67, 168.29. Mass analysis (*m/z*, %): 557 (M^+^, Br-81, 32.05%), 555 (M^+^, Br-79, 32.37%). Analysis for C_23_H_18_BrN_5_O_5_S (556.39): Calculated: C, 49.65; H, 3.26; N, 12.59%. Found: C, 49.49; H, 3.21; N, 12.65%.

### Methyl 5-oxo-4-(2-(4-(N-(pyridin-3-yl)sulfamoyl)phenyl)hydrazono)-2-(thiophen-2-yl)-4,5-dihydro-1H-pyrrole-3-carboxylate (4f)

 Orange powder, recrystallized from EtOH, yield = 83%, m.p. = 265–267 °C. IR (KBr): 3425, 3227, 3167, 3120 (N–H), 1697, 1657 cm^−1^ (C=O), 1357, 1142 (SO_2_). ^1^H NMR: δ 3.75 (s, 3H, –OCH_3_), 7.12 (d, *J* = 4.50 Hz, 1H, pyridine-H), 7.38–7.40 (m, 1H, thiophene-H), 7.67 (d, *J* = 8.50 Hz, 2H, Ar–H), 7.73–7.80 (m, 2H, pyridine-H), 7.86 (d, *J* = 8.50 Hz, 2H, Ar–H), 7.95–7.99 (m, 2H, thiophene-H), 8.10 (s, 1H, pyridine-H), 11.29 (s, 1H, N–H), 12.70 (s, 1H, N–H), 13.16 ppm (s, 1H, N–H). ^13^C NMR: δ 51.03, 107.13, 115.29 (2C), 123.56, 124.80, 127.03, 129.35, 130.06, 131.62 (2C), 131.93, 135.18, 138.76, 139.20, 140.81, 147.39, 149.17, 152.04, 157.49, 166.19. Mass analysis (*m/z*, %): 483 (25.27%). Analysis for C_21_H_17_N_5_O_5_S_2_ (483.52): Calculated: C, 52.17; H, 3.54; N, 14.48%. Found: C, 52.08; H, 3.58; N, 14.37%.

## DFT computational calculations

The synthesized derivatives were geometrically optimized in gas phase at DFT/B3LYP/6-311+ + G(d,p)^62–64^ implemented in Gaussian 09W program^65^ and the structural and electronic outcomes was explored using GaussView software^66^.

## Antibacterial evaluation

The antibacterial effectiveness of the newly prepared sulfonamide hybrids toward ATCC bacterial strains, clinically isolated Gram+ and Gram− bacteria are used. The assigning of antibacterial effectiveness was performed via agar disk diffusion. The results established several antibacterial effectiveness of the newly synthesized sulfonamide derivatives against bacteria determined in this study. The newly synthesized sulfonamide derivatives displayed antibacterial activities against four ATCC bacterial strains *B. subtilis* ATCC 6633, *S. aureus* ATCC 25923, *S. typhimurium* ATCC 14028, and *E. coli* ATCC 25922^67^. Meanwhile, minimum inhibitory concentration (MIC) was established as the lowest quantity that totally blocks observable bacterial growth. As a result, one loop of the MIC solution that seemed optically clear was grown on agar plates and cultured at 37 °C for 20 h^68^.

## Molecular docking

Starting with the X-ray structure, theoretical docking simulation was run to explore the bindings of the newly prepared sulfonamide analogues' ligand structures against two diverse proteins a wild type enzyme DHPS (Dihydroptorate Synthase of Versinia pestis, PDB: ID 3TZF)^69^ and *S. aureus* dihydropteroate synthase (saDHPS) which was represented by (PDB ID: 6CLV)strain rummage-sale in this work^51^.

## In silico ADME analysis

Using the publicly available Swiss ADME programme, we investigated the in silico physicochemical and pharmacokinetic features of newly synthesised sulfonamide hybrids with decreased MIC values (16, 18, 20, 21, and 23 µg/mL). This software provides access to a pool of fast yet modest predictive models that use simple molecular and physicochemical descriptors, such as molecular weight^18^, the count of specific types of bonds (the numbers of heavy atoms, aromatic heavy atoms, rotatable bonds, hydrogen-bond acceptors, hydrogen-bond donors), topological polar surface area (TPSA), and several others, all of which are important determinants in predicting good drug/lead-like^70^.

## Conclusion

Finally, by inventing and synthesizing a new series of 5-oxo-2-phenyl-4-(arylsulfamoyl)phenyl)hydrazono)-4,5-dihydro-*1H*-pyrrole-3-carboxylate hybrids **4a-f**, this study addressed the difficulty of sulfonamide resistance in antibacterial therapy. Through structural alterations, the goal was to overcome resistance mechanisms and find possible treatment options. The chemical configurations of the prepared hybrids were confirmed using spectroscopic methods. The DFT calculations were rummage-sale to clarify the FMO’s construction and energies of the explored hybrids and discovered that they have low HOMO–LUMO energy gap (ΔE_H-L_), 1.68–1.75 eV, and may be sorted as **4b** < **4e** < **4c** < **4a** < **4f** < **4d**.

The antibacterial activity of sulfonamide hybrid **4a** against *S. typhimurium* was exceptional, with IZD of 15 mm and MIC of 19.24 µg/mL. Furthermore, it inhibited *E. coli* significantly, with an IZD of 19 mm and MIC of 11.31 µg/mL, surpassing the reference sulfamethazole. Hybrid **4d** also demonstrated significant antibacterial activity over *S. typhimurium*, with by IZD of 16 mm and MIC of 19.24 µg/mL, as well as increased inhibition against *E. coli*. Furthermore, using the PDB codes 3TZF and 6CLV, docking stimulation technique were achieved to analyze the binding interactions of the produced sulfonamide hybrids. When compared to other hybrids, hybrids **4e** and **4c** had the highest binding score, indicating robust interactions with the target proteins, respectively. The findings of this study show that these newly synthesized hybrids have the potential to be efficient antibacterial agents against resistant pathogens. Their improved antibacterial activity, as revealed by both experimental testing and molecular docking, provides important insights for the further development and optimization of potential treatment options. This discovery contributes to current efforts to battle bacterial infections by overcoming sulfonamide resistance and establishing a structure–activity link, paving the door for the development of more potent antibacterial drug.

### Supplementary Information

Supplementary Information.

## Data Availability

All data generated or analyzed during this study are included in this article [and its supplementary information file.
