# Supplementary material for: Synthesis, molecular modelling, and antibacterial evaluation of new sulfonamide-dyes based pyrrole compounds
Source: Sci Rep. 2024 May 14;14:10973. doi: 10.1038/s41598-024-60908-8 (PMC11094129; doi:10.1038/s41598-024-60908-8)
Supplement: Supplementary file 1 — Supplementary Information. [file 41598_2024_60908_MOESM1_ESM.docx]

Supporting Information

**DFT studies**

**Table S1**. The DFT dihedral angle data of the investigated compounds.

|  | **4a** |  | **4d** |  | **4c** |
| --- | --- | --- | --- | --- | --- |
| C(12)-O(31)-C(33)-C(34) | -82.8 | C(12)-O(32)-C(34)-C(35) | -82.2 | C(27)-C(28)-S(29)-C(1) | 1.4 |
| C(26)-C(27)-N(28)-C(24) | -0.9 | C(29)-C(30)-C(31)-C(24) | -1.3 | C(26)-C(27)-C(28)-S(29) | -1.3 |
| S(25)-C(26)-C(27)-N(28) | 1.7 | N(28)-C(29)-C(30)-C(31) | 1.2 | C(1)-C(26)-C(27)-C(28) | 0.5 |
| C(24)-S(25)-C(26)-C(27) | -1.5 | C(27)-N(28)-C(29)-C(30) | 0.3 | C(21)-C(22)-N(23)-C(19) | -0.7 |
| N(23)-C(24)-N(28)-C(27) | -179.8 | C(24)-C(27)-N(28)-C(29) | -1.6 | S(20)-C(21)-C(22)-N(23) | 1.6 |
| S(25)-C(24)-N(28)-C(27) | -0.3 | N(23)-C(24)-C(31)-C(30) | -178.6 | C(19)-S(20)-C(21)-C(22) | -1.4 |
| N(23)-C(24)-S(25)-C(26) | -179.5 | C(27)-C(24)-C(31)-C(30) | 0.1 | N(18)-C(19)-N(23)-C(22) | -179.8 |
| N(28)-C(24)-S(25)-C(26) | 1.1 | N(23)-C(24)-C(27)-N(28) | -179.9 | S(20)-C(19)-N(23)-C(22) | -0.5 |
| S(22)-N(23)-C(24)-S(25) | -142.1 | C(31)-C(24)-C(27)-N(28) | 1.4 | N(18)-C(19)-S(20)-C(21) | -179.5 |
| S(22)-N(23)-C(24)-N(28) | 37.3 | S(22)-N(23)-C(24)-C(27) | 52.5 | N(23)-C(19)-S(20)-C(21) | 1.1 |
| H(46)-N(23)-C(24)-S(25) | 4.5 | S(22)-N(23)-C(24)-C(31) | -128.9 | S(17)-N(18)-C(19)-S(20) | -137.1 |
| H(46)-N(23)-C(24)-N(28) | -176.1 | H(47)-N(23)-C(24)-C(27) | -172.4 | S(17)-N(18)-C(19)-N(23) | 42.2 |
| C(19)-S(22)-N(23)-C(24) | -87.3 | H(47)-N(23)-C(24)-C(31) | 6.3 | H(39)-N(18)-C(19)-S(20) | -5.3 |
| C(19)-S(22)-N(23)-H(46) | 123.5 | C(19)-S(22)-N(23)-C(24) | 54.7 | H(39)-N(18)-C(19)-N(23) | 174.0 |
| O(29)-S(22)-N(23)-C(24) | 159.9 | C(19)-S(22)-N(23)-H(47) | -82.8 | C(14)-S(17)-N(18)-C(19) | 161.9 |
| O(29)-S(22)-N(23)-H(46) | 10.6 | O(25)-S(22)-N(23)-C(24) | -59.5 | C(14)-S(17)-N(18)-H(39) | 27.7 |
| O(30)-S(22)-N(23)-C(24) | 28.8 | O(25)-S(22)-N(23)-H(47) | 163.0 | O(24)-S(17)-N(18)-C(19) | 48.9 |
| O(30)-S(22)-N(23)-H(46) | -120.5 | O(26)-S(22)-N(23)-C(24) | 168.9 | O(24)-S(17)-N(18)-H(39) | -85.3 |
| C(19)-C(20)-C(21)-C(16) | 0.0 | O(26)-S(22)-N(23)-H(47) | 31.4 | O(25)-S(17)-N(18)-C(19) | -85.0 |
| C(18)-C(19)-S(22)-N(23) | 80.8 | C(19)-C(20)-C(21)-C(16) | 0.2 | O(25)-S(17)-N(18)-H(39) | 140.8 |
| C(18)-C(19)-S(22)-O(29) | -171.6 | C(18)-C(19)-S(22)-N(23) | -87.9 | C(14)-C(15)-C(16)-C(11) | 0.3 |
| C(18)-C(19)-S(22)-O(30) | -34.7 | C(18)-C(19)-S(22)-O(25) | 25.2 | C(13)-C(14)-S(17)-N(18) | 85.6 |
| C(20)-C(19)-S(22)-N(23) | -100.9 | C(18)-C(19)-S(22)-O(26) | 162.2 | C(13)-C(14)-S(17)-O(24) | -161.6 |
| C(20)-C(19)-S(22)-O(29) | 6.7 | C(20)-C(19)-S(22)-N(23) | 89.3 | C(13)-C(14)-S(17)-O(25) | -25.6 |
| C(20)-C(19)-S(22)-O(30) | 143.6 | C(20)-C(19)-S(22)-O(25) | -157.6 | C(15)-C(14)-S(17)-N(18) | -95.4 |
| C(18)-C(19)-C(20)-C(21) | -0.2 | C(20)-C(19)-S(22)-O(26) | -20.6 | C(15)-C(14)-S(17)-O(24) | 17.4 |
| S(22)-C(19)-C(20)-C(21) | -178.5 | C(18)-C(19)-C(20)-C(21) | 0.7 | C(15)-C(14)-S(17)-O(25) | 153.3 |
| C(17)-C(18)-C(19)-C(20) | 0.3 | S(22)-C(19)-C(20)-C(21) | -176.4 | C(13)-C(14)-C(15)-C(16) | -1.2 |
| C(17)-C(18)-C(19)-S(22) | 178.6 | C(17)-C(18)-C(19)-C(20) | -0.9 | S(17)-C(14)-C(15)-C(16) | 179.9 |
| C(16)-C(17)-C(18)-C(19) | -0.2 | C(17)-C(18)-C(19)-S(22) | 176.2 | C(12)-C(13)-C(14)-C(15) | 1.2 |
| N(15)-C(16)-C(21)-C(20) | 180.0 | C(16)-C(17)-C(18)-C(19) | 0.2 | C(12)-C(13)-C(14)-S(17) | -179.9 |
| C(17)-C(16)-C(21)-C(20) | 0.1 | N(15)-C(16)-C(21)-C(20) | 179.5 | C(11)-C(12)-C(13)-C(14) | -0.3 |
| N(15)-C(16)-C(17)-C(18) | -179.9 | C(17)-C(16)-C(21)-C(20) | -0.9 | N(10)-C(11)-C(16)-C(15) | -180.0 |
| C(21)-C(16)-C(17)-C(18) | 0.0 | N(15)-C(16)-C(17)-C(18) | -179.7 | C(12)-C(11)-C(16)-C(15) | 0.6 |
| N(14)-N(15)-C(16)-C(17) | -179.0 | C(21)-C(16)-C(17)-C(18) | 0.7 | N(10)-C(11)-C(12)-C(13) | 180.0 |
| N(14)-N(15)-C(16)-C(21) | 1.1 | N(14)-N(15)-C(16)-C(17) | 179.0 | C(16)-C(11)-C(12)-C(13) | -0.5 |
| H(41)-N(15)-C(16)-C(17) | 0.0 | N(14)-N(15)-C(16)-C(21) | -1.4 | N(9)-N(10)-C(11)-C(12) | -178.7 |
| H(41)-N(15)-C(16)-C(21) | -179.8 | H(42)-N(15)-C(16)-C(17) | -2.1 | N(9)-N(10)-C(11)-C(16) | 1.9 |
| C(10)-N(14)-N(15)-C(16) | -179.3 | H(42)-N(15)-C(16)-C(21) | 177.6 | H(34)-N(10)-C(11)-C(12) | -0.8 |
| C(10)-N(14)-N(15)-H(41) | 1.7 | C(10)-N(14)-N(15)-C(16) | -179.5 | H(34)-N(10)-C(11)-C(16) | 179.8 |
| C(11)-C(12)-O(31)-C(33) | 179.5 | C(10)-N(14)-N(15)-H(42) | 1.5 | C(5)-N(9)-N(10)-C(11) | -179.9 |
| O(32)-C(12)-O(31)-C(33) | 0.1 | C(11)-C(12)-O(32)-C(34) | 179.0 | C(5)-N(9)-N(10)-H(34) | 2.2 |
| C(7)-C(11)-C(12)-O(31) | 1.5 | O(33)-C(12)-O(32)-C(34) | -0.4 | C(6)-C(7)-O(30)-C(32) | 178.8 |
| C(7)-C(11)-C(12)-O(32) | -179.2 | C(7)-C(11)-C(12)-O(32) | 1.9 | O(31)-C(7)-O(30)-C(32) | -1.2 |
| C(10)-C(11)-C(12)-O(31) | -170.4 | C(7)-C(11)-C(12)-O(33) | -178.8 | C(2)-C(6)-C(7)-O(30) | 170.8 |
| C(10)-C(11)-C(12)-O(32) | 8.9 | C(10)-C(11)-C(12)-O(32) | -169.9 | C(2)-C(6)-C(7)-O(31) | -9.2 |
| C(9)-C(10)-N(14)-N(15) | 179.9 | C(10)-C(11)-C(12)-O(33) | 9.4 | C(5)-C(6)-C(7)-O(30) | -8.7 |
| C(11)-C(10)-N(14)-N(15) | 0.6 | C(9)-C(10)-N(14)-N(15) | 179.6 | C(5)-C(6)-C(7)-O(31) | 171.3 |
| C(9)-C(10)-C(11)-C(7) | -0.8 | C(11)-C(10)-N(14)-N(15) | 0.0 | C(4)-C(5)-N(9)-N(10) | -180.0 |
| C(9)-C(10)-C(11)-C(12) | 172.3 | C(9)-C(10)-C(11)-C(7) | -0.5 | C(6)-C(5)-N(9)-N(10) | 1.5 |
| N(14)-C(10)-C(11)-C(7) | 178.5 | C(9)-C(10)-C(11)-C(12) | 172.6 | C(4)-C(5)-C(6)-C(2) | 0.9 |
| N(14)-C(10)-C(11)-C(12) | -8.4 | N(14)-C(10)-C(11)-C(7) | 179.1 | C(4)-C(5)-C(6)-C(7) | -179.5 |
| N(8)-C(9)-C(10)-C(11) | -0.8 | N(14)-C(10)-C(11)-C(12) | -7.8 | N(9)-C(5)-C(6)-C(2) | 179.5 |
| N(8)-C(9)-C(10)-N(14) | 179.7 | N(8)-C(9)-C(10)-C(11) | -1.0 | N(9)-C(5)-C(6)-C(7) | -0.9 |
| O(13)-C(9)-C(10)-C(11) | 178.2 | N(8)-C(9)-C(10)-N(14) | 179.3 | N(3)-C(4)-C(5)-C(6) | -1.7 |
| O(13)-C(9)-C(10)-N(14) | -1.2 | O(13)-C(9)-C(10)-C(11) | 177.9 | N(3)-C(4)-C(5)-N(9) | 179.3 |
| C(7)-N(8)-C(9)-C(10) | 2.311 | O(13)-C(9)-C(10)-N(14) | -1.8 | O(8)-C(4)-C(5)-C(6) | 177.6 |
| C(7)-N(8)-C(9)-O(13) | -176.835 | C(7)-N(8)-C(9)-C(10) | 2.3 | O(8)-C(4)-C(5)-N(9) | -1.4 |
| H(40)-N(8)-C(9)-C(10) | 170.38 | C(7)-N(8)-C(9)-O(13) | -176.7 | C(2)-N(3)-C(4)-C(5) | 1.9 |
| H(40)-N(8)-C(9)-O(13) | -8.767 | H(41)-N(8)-C(9)-C(10) | 170.6 | C(2)-N(3)-C(4)-O(8) | -177.4 |
| C(3)-C(7)-C(11)-C(10) | -177.672 | H(41)-N(8)-C(9)-O(13) | -8.4 | H(33)-N(3)-C(4)-C(5) | 171.9 |
| C(3)-C(7)-C(11)-C(12) | 9.052 | C(3)-C(7)-C(11)-C(10) | -178.2 | H(33)-N(3)-C(4)-O(8) | -7.4 |
| N(8)-C(7)-C(11)-C(10) | 2.256 | C(3)-C(7)-C(11)-C(12) | 8.6 | C(1)-C(2)-C(6)-C(5) | 177.4 |
| N(8)-C(7)-C(11)-C(12) | -171.02 | N(8)-C(7)-C(11)-C(10) | 1.967 | C(1)-C(2)-C(6)-C(7) | -2.2 |
| C(3)-C(7)-N(8)-C(9) | 176.967 | N(8)-C(7)-C(11)-C(12) | -171.238 | N(3)-C(2)-C(6)-C(5) | 0.3 |
| C(3)-C(7)-N(8)-H(40) | 9.186 | C(3)-C(7)-N(8)-C(9) | 177.358 | N(3)-C(2)-C(6)-C(7) | -179.4 |
| C(11)-C(7)-N(8)-C(9) | -2.974 | C(3)-C(7)-N(8)-H(41) | 9.341 | C(1)-C(2)-N(3)-C(4) | -179.2 |
| C(11)-C(7)-N(8)-H(40) | -170.755 | C(11)-C(7)-N(8)-C(9) | -2.797 | C(1)-C(2)-N(3)-H(33) | 11.3 |
| C(4)-C(5)-C(6)-C(1) | -0.111 | C(11)-C(7)-N(8)-H(41) | -170.814 | C(6)-C(2)-N(3)-C(4) | -1.4 |
| C(3)-C(4)-C(5)-C(6) | 1.09 | C(4)-C(5)-C(6)-C(1) | -0.14 | C(6)-C(2)-N(3)-H(33) | -171.0 |
| C(2)-C(3)-C(7)-N(8) | -134.879 | C(3)-C(4)-C(5)-C(6) | 1.121 | C(2)-C(1)-S(29)-C(28) | 176.0 |
| C(2)-C(3)-C(7)-C(11) | 45.045 | C(2)-C(3)-C(7)-N(8) | -135.335 | C(26)-C(1)-S(29)-C(28) | -1.0 |
| C(4)-C(3)-C(7)-N(8) | 40.785 | C(2)-C(3)-C(7)-C(11) | 44.866 | C(2)-C(1)-C(26)-C(27) | -176.7 |
| C(4)-C(3)-C(7)-C(11) | -139.292 | C(4)-C(3)-C(7)-N(8) | 40.368 | S(29)-C(1)-C(26)-C(27) | 0.5 |
| C(2)-C(3)-C(4)-C(5) | -1.294 | C(4)-C(3)-C(7)-C(11) | -139.432 | C(26)-C(1)-C(2)-N(3) | 11.8 |
| C(7)-C(3)-C(4)-C(5) | -177.08 | C(2)-C(3)-C(4)-C(5) | -1.315 | C(26)-C(1)-C(2)-C(6) | -165.2 |
| C(1)-C(2)-C(3)-C(4) | 0.53 | C(7)-C(3)-C(4)-C(5) | -177.145 | S(29)-C(1)-C(2)-N(3) | -164.9 |
| C(1)-C(2)-C(3)-C(7) | 176.186 | C(1)-C(2)-C(3)-C(4) | 0.538 | S(29)-C(1)-C(2)-C(6) | 18.1 |
| C(2)-C(1)-C(6)-C(5) | -0.651 | C(1)-C(2)-C(3)-C(7) | 176.228 |  |  |
| C(6)-C(1)-C(2)-C(3) | 0.436 | C(2)-C(1)-C(6)-C(5) | -0.634 |  |  |
|  |  | C(6)-C(1)-C(2)-C(3) | 0.43 |  |  |
|  | **4f** |  | **4b** |  | **4e** |
| C(28)-C(29)-S(30)-C(1) | -1.4 | C(26)-C(27)-N(28)-C(24) | 0.0 | C(29)-C(30)-C(31)-C(24) | 0.0 |
| C(27)-C(28)-C(29)-S(30) | 1.3 | S(25)-C(26)-C(27)-N(28) | -0.6 | N(28)-C(29)-C(30)-C(31) | -0.3 |
| C(1)-C(27)-C(28)-C(29) | -0.5 | C(24)-S(25)-C(26)-C(27) | 0.7 | C(27)-N(28)-C(29)-C(30) | 0.2 |
| C(24)-C(25)-C(26)-C(19) | 1.1 | N(23)-C(24)-N(28)-C(27) | -177.8 | C(24)-C(27)-N(28)-C(29) | 0.3 |
| N(23)-C(24)-C(25)-C(26) | -0.6 | S(25)-C(24)-N(28)-C(27) | 0.5 | N(23)-C(24)-C(31)-C(30) | -177.7 |
| C(22)-N(23)-C(24)-C(25) | -0.3 | N(23)-C(24)-S(25)-C(26) | 177.7 | C(27)-C(24)-C(31)-C(30) | 0.4 |
| C(19)-C(22)-N(23)-C(24) | 0.8 | N(28)-C(24)-S(25)-C(26) | -0.7 | N(23)-C(24)-C(27)-N(28) | 177.5 |
| N(18)-C(19)-C(26)-C(25) | 175.9 | S(22)-N(23)-C(24)-S(25) | 152.8 | C(31)-C(24)-C(27)-N(28) | -0.6 |
| C(22)-C(19)-C(26)-C(25) | -0.7 | S(22)-N(23)-C(24)-N(28) | -29.0 | S(22)-N(23)-C(24)-C(27) | 18.4 |
| N(18)-C(19)-C(22)-N(23) | -176.8 | H(45)-N(23)-C(24)-S(25) | 19.0 | S(22)-N(23)-C(24)-C(31) | -163.6 |
| C(26)-C(19)-C(22)-N(23) | -0.3 | H(45)-N(23)-C(24)-N(28) | -162.8 | H(46)-N(23)-C(24)-C(27) | 171.5 |
| S(17)-N(18)-C(19)-C(22) | -62.9 | C(19)-S(22)-N(23)-C(24) | -176.4 | H(46)-N(23)-C(24)-C(31) | -10.4 |
| S(17)-N(18)-C(19)-C(26) | 120.6 | C(19)-S(22)-N(23)-H(45) | -40.5 | C(19)-S(22)-N(23)-C(24) | -80.7 |
| H(40)-N(18)-C(19)-C(22) | 168.1 | O(29)-S(22)-N(23)-C(24) | 70.0 | C(19)-S(22)-N(23)-H(46) | 124.3 |
| H(40)-N(18)-C(19)-C(26) | -8.4 | O(29)-S(22)-N(23)-H(45) | -154.2 | O(25)-S(22)-N(23)-C(24) | 165.6 |
| C(14)-S(17)-N(18)-C(19) | 151.4 | O(30)-S(22)-N(23)-C(24) | -64.2 | O(25)-S(22)-N(23)-H(46) | 10.7 |
| C(14)-S(17)-N(18)-H(40) | -77.6 | O(30)-S(22)-N(23)-H(45) | 71.6 | O(26)-S(22)-N(23)-C(24) | 33.9 |
| O(20)-S(17)-N(18)-C(19) | 36.5 | C(19)-C(20)-C(21)-C(16) | 0.0 | O(26)-S(22)-N(23)-H(46) | -121.1 |
| O(20)-S(17)-N(18)-H(40) | 167.6 | C(18)-C(19)-S(22)-N(23) | -68.4 | C(19)-C(20)-C(21)-C(16) | -0.3 |
| O(21)-S(17)-N(18)-C(19) | -96.1 | C(18)-C(19)-S(22)-O(29) | 42.4 | C(18)-C(19)-S(22)-N(23) | -70.4 |
| O(21)-S(17)-N(18)-H(40) | 35.0 | C(18)-C(19)-S(22)-O(30) | 178.2 | C(18)-C(19)-S(22)-O(25) | 37.5 |
| C(14)-C(15)-C(16)-C(11) | 0.5 | C(20)-C(19)-S(22)-N(23) | 115.1 | C(18)-C(19)-S(22)-O(26) | 173.1 |
| C(13)-C(14)-S(17)-N(18) | 120.1 | C(20)-C(19)-S(22)-O(29) | -134.2 | C(20)-C(19)-S(22)-N(23) | 111.3 |
| C(13)-C(14)-S(17)-O(20) | -128.9 | C(20)-C(19)-S(22)-O(30) | 1.7 | C(20)-C(19)-S(22)-O(25) | -140.9 |
| C(13)-C(14)-S(17)-O(21) | 5.8 | C(18)-C(19)-C(20)-C(21) | 1.6 | C(20)-C(19)-S(22)-O(26) | -5.2 |
| C(15)-C(14)-S(17)-N(18) | -62.4 | S(22)-C(19)-C(20)-C(21) | 178.0 | C(18)-C(19)-C(20)-C(21) | 1.1 |
| C(15)-C(14)-S(17)-O(20) | 48.6 | C(17)-C(18)-C(19)-C(20) | -2.5 | S(22)-C(19)-C(20)-C(21) | 179.4 |
| C(15)-C(14)-S(17)-O(21) | -176.7 | C(17)-C(18)-C(19)-S(22) | -178.9 | C(17)-C(18)-C(19)-C(20) | -1.1 |
| C(13)-C(14)-C(15)-C(16) | -0.7 | C(16)-C(17)-C(18)-C(19) | 1.7 | C(17)-C(18)-C(19)-S(22) | -179.4 |
| S(17)-C(14)-C(15)-C(16) | -178.0 | N(15)-C(16)-C(21)-C(20) | 178.9 | C(16)-C(17)-C(18)-C(19) | 0.4 |
| C(12)-C(13)-C(14)-C(15) | 0.4 | C(17)-C(16)-C(21)-C(20) | -0.7 | N(15)-C(16)-C(21)-C(20) | 179.9 |
| C(12)-C(13)-C(14)-S(17) | 177.8 | N(15)-C(16)-C(17)-C(18) | -179.8 | C(17)-C(16)-C(21)-C(20) | -0.4 |
| C(11)-C(12)-C(13)-C(14) | 0.0 | C(21)-C(16)-C(17)-C(18) | -0.2 | N(15)-C(16)-C(17)-C(18) | -179.9 |
| N(10)-C(11)-C(16)-C(15) | -180.0 | N(14)-N(15)-C(16)-C(17) | 179.7 | C(21)-C(16)-C(17)-C(18) | 0.4 |
| C(12)-C(11)-C(16)-C(15) | -0.1 | N(14)-N(15)-C(16)-C(21) | 0.1 | N(14)-N(15)-C(16)-C(17) | -178.8 |
| N(10)-C(11)-C(12)-C(13) | 179.7 | H(40)-N(15)-C(16)-C(17) | -4.9 | N(14)-N(15)-C(16)-C(21) | 0.9 |
| C(16)-C(11)-C(12)-C(13) | -0.1 | H(40)-N(15)-C(16)-C(21) | 175.5 | H(41)-N(15)-C(16)-C(17) | -0.1 |
| N(9)-N(10)-C(11)-C(12) | 179.9 | C(10)-N(14)-N(15)-C(16) | 179.3 | H(41)-N(15)-C(16)-C(21) | 179.6 |
| N(9)-N(10)-C(11)-C(16) | -0.3 | C(10)-N(14)-N(15)-H(40) | 3.7 | C(10)-N(14)-N(15)-C(16) | -179.5 |
| H(35)-N(10)-C(11)-C(12) | 2.7 | C(11)-C(12)-O(32)-C(33) | 178.0 | C(10)-N(14)-N(15)-H(41) | 1.7 |
| H(35)-N(10)-C(11)-C(16) | -177.4 | O(34)-C(12)-O(32)-C(33) | -1.1 | C(11)-C(12)-O(33)-C(35) | 179.5 |
| C(5)-N(9)-N(10)-C(11) | 179.9 | C(7)-C(11)-C(12)-O(32) | -2.1 | O(34)-C(12)-O(33)-C(35) | 0.4 |
| C(5)-N(9)-N(10)-H(35) | -2.9 | C(7)-C(11)-C(12)-O(34) | 177.0 | C(7)-C(11)-C(12)-O(33) | 1.3 |
| C(6)-C(7)-O(31)-C(33) | -179.2 | C(10)-C(11)-C(12)-O(32) | -174.4 | C(7)-C(11)-C(12)-O(34) | -179.7 |
| O(32)-C(7)-O(31)-C(33) | 0.9 | C(10)-C(11)-C(12)-O(34) | 4.7 | C(10)-C(11)-C(12)-O(33) | -170.5 |
| C(2)-C(6)-C(7)-O(31) | -170.0 | C(9)-C(10)-N(14)-N(15) | 179.8 | C(10)-C(11)-C(12)-O(34) | 8.6 |
| C(2)-C(6)-C(7)-O(32) | 9.9 | C(11)-C(10)-N(14)-N(15) | 0.4 | C(9)-C(10)-N(14)-N(15) | -179.7 |
| C(5)-C(6)-C(7)-O(31) | 9.0 | C(9)-C(10)-C(11)-C(7) | -0.5 | C(11)-C(10)-N(14)-N(15) | 0.5 |
| C(5)-C(6)-C(7)-O(32) | -171.1 | C(9)-C(10)-C(11)-C(12) | 173.1 | C(9)-C(10)-C(11)-C(7) | -0.6 |
| C(4)-C(5)-N(9)-N(10) | -179.4 | N(14)-C(10)-C(11)-C(7) | 179.0 | C(9)-C(10)-C(11)-C(12) | 172.5 |
| C(6)-C(5)-N(9)-N(10) | -1.4 | N(14)-C(10)-C(11)-C(12) | -7.4 | N(14)-C(10)-C(11)-C(7) | 179.2 |
| C(4)-C(5)-C(6)-C(2) | -0.8 | N(8)-C(9)-C(10)-C(11) | -0.7 | N(14)-C(10)-C(11)-C(12) | -7.7 |
| C(4)-C(5)-C(6)-C(7) | -179.9 | N(8)-C(9)-C(10)-N(14) | 179.8 | N(8)-C(9)-C(10)-C(11) | -1.1 |
| N(9)-C(5)-C(6)-C(2) | -178.9 | O(13)-C(9)-C(10)-C(11) | 178.5 | N(8)-C(9)-C(10)-N(14) | 179.1 |
| N(9)-C(5)-C(6)-C(7) | 2.0 | O(13)-C(9)-C(10)-N(14) | -1.1 | O(13)-C(9)-C(10)-C(11) | 178.0 |
| N(3)-C(4)-C(5)-C(6) | 1.6 | C(7)-N(8)-C(9)-C(10) | 1.6 | O(13)-C(9)-C(10)-N(14) | -1.8 |
| N(3)-C(4)-C(5)-N(9) | -179.9 | C(7)-N(8)-C(9)-O(13) | -177.6 | C(7)-N(8)-C(9)-C(10) | 2.5 |
| O(8)-C(4)-C(5)-C(6) | -177.5 | H(39)-N(8)-C(9)-C(10) | 173.5 | C(7)-N(8)-C(9)-O(13) | -176.7 |
| O(8)-C(4)-C(5)-N(9) | 1.1 | H(39)-N(8)-C(9)-O(13) | -5.7 | H(40)-N(8)-C(9)-C(10) | 170.1 |
| C(2)-N(3)-C(4)-C(5) | -1.8 | C(3)-C(7)-C(11)-C(10) | -179.9 | H(40)-N(8)-C(9)-O(13) | -9.1 |
| C(2)-N(3)-C(4)-O(8) | 177.3 | C(3)-C(7)-C(11)-C(12) | 6.6 | C(3)-C(7)-C(11)-C(10) | -178.2 |
| H(34)-N(3)-C(4)-C(5) | -172.1 | N(8)-C(7)-C(11)-C(10) | 1.5 | C(3)-C(7)-C(11)-C(12) | 8.7 |
| H(34)-N(3)-C(4)-O(8) | 7.1 | N(8)-C(7)-C(11)-C(12) | -172.0 | N(8)-C(7)-C(11)-C(10) | 2.1 |
| C(1)-C(2)-C(6)-C(5) | -177.3 | C(3)-C(7)-N(8)-C(9) | 179.1 | N(8)-C(7)-C(11)-C(12) | -171.0 |
| C(1)-C(2)-C(6)-C(7) | 1.9 | C(3)-C(7)-N(8)-H(39) | 7.4 | C(3)-C(7)-N(8)-C(9) | 177.3 |
| N(3)-C(2)-C(6)-C(5) | -0.3 | C(11)-C(7)-N(8)-C(9) | -2.0 | C(3)-C(7)-N(8)-H(40) | 10.0 |
| N(3)-C(2)-C(6)-C(7) | 178.9 | C(11)-C(7)-N(8)-H(39) | -173.7 | C(11)-C(7)-N(8)-C(9) | -3.0 |
| C(1)-C(2)-N(3)-C(4) | 179.0 | C(4)-C(5)-C(6)-C(1) | -0.3 | C(11)-C(7)-N(8)-H(40) | -170.3 |
| C(1)-C(2)-N(3)-H(34) | -11.1 | C(4)-C(5)-C(6)-Br(31) | -179.9 | C(4)-C(5)-C(6)-C(1) | 0.0 |
| C(6)-C(2)-N(3)-C(4) | 1.4 | C(3)-C(4)-C(5)-C(6) | 1.3 | C(4)-C(5)-C(6)-Br(32) | 179.7 |
| C(6)-C(2)-N(3)-H(34) | 171.2 | C(2)-C(3)-C(7)-N(8) | -133.2 | C(3)-C(4)-C(5)-C(6) | 1.1 |
| C(2)-C(1)-S(30)-C(29) | -176.0 | C(2)-C(3)-C(7)-C(11) | 48.3 | C(2)-C(3)-C(7)-N(8) | -135.8 |
| C(27)-C(1)-S(30)-C(29) | 1.0 | C(4)-C(3)-C(7)-N(8) | 43.3 | C(2)-C(3)-C(7)-C(11) | 44.5 |
| C(2)-C(1)-C(27)-C(28) | 176.7 | C(4)-C(3)-C(7)-C(11) | -135.2 | C(4)-C(3)-C(7)-N(8) | 40.0 |
| S(30)-C(1)-C(27)-C(28) | -0.5 | C(2)-C(3)-C(4)-C(5) | -1.3 | C(4)-C(3)-C(7)-C(11) | -139.7 |
| C(27)-C(1)-C(2)-N(3) | -11.851 | C(7)-C(3)-C(4)-C(5) | -178.0 | C(2)-C(3)-C(4)-C(5) | -1.4 |
| C(27)-C(1)-C(2)-C(6) | 165.019 | C(1)-C(2)-C(3)-C(4) | 0.3 | C(7)-C(3)-C(4)-C(5) | -177.3 |
| S(30)-C(1)-C(2)-N(3) | 164.836 | C(1)-C(2)-C(3)-C(7) | 176.8 | C(1)-C(2)-C(3)-C(4) | 0.6 |
| S(30)-C(1)-C(2)-C(6) | -18.294 | C(2)-C(1)-C(6)-C(5) | -0.7 | C(1)-C(2)-C(3)-C(7) | 176.4 |
|  |  | C(2)-C(1)-C(6)-Br(31) | 178.9 | C(2)-C(1)-C(6)-C(5) | -0.8 |
|  |  | C(6)-C(1)-C(2)-C(3) | 0.7 | C(2)-C(1)-C(6)-Br(32) | 179.5 |
|  |  |  |  | C(6)-C(1)-C(2)-C(3) | 0.5 |

**Table S2**. The DFT bond length data of the investigated compounds.

|  | **4a** | **Std** |  | **4d** | **Std** |  | **4c** | **Std** |
| --- | --- | --- | --- | --- | --- | --- | --- | --- |
| C(33)-C(34) | 1.50 | 1.51 | C(34)-C(35) | 1.50 | 1.51 | O(30)-C(32) | 1.43 | 1.40 |
| O(31)-C(33) | 1.43 | 1.39 | O(32)-C(34) | 1.44 | 1.39 | C(28)-S(29) | 1.70 | 1.66 |
| C(27)-N(28) | 1.36 | 1.36 | C(30)-C(31) | 1.39 | 1.42 | C(27)-C(28) | 1.38 | 1.42 |
| C(26)-C(27) | 1.37 | 1.42 | C(29)-C(30) | 1.39 | 1.42 | C(26)-C(27) | 1.40 | 1.42 |
| S(25)-C(26) | 1.72 | 1.66 | N(28)-C(29) | 1.33 | 1.36 | C(22)-N(23) | 1.36 | 1.36 |
| C(24)-N(28) | 1.30 | 1.36 | C(27)-N(28) | 1.33 | 1.36 | C(21)-C(22) | 1.37 | 1.42 |
| C(24)-S(25) | 1.75 | 1.66 | C(24)-C(31) | 1.40 | 1.42 | S(20)-C(21) | 1.72 | 1.66 |
| N(23)-H(46) | 1.03 | 1.05 | C(24)-C(27) | 1.40 | 1.42 | C(19)-N(23) | 1.30 | 1.36 |
| N(23)-C(24) | 1.38 | 1.46 | N(23)-H(47) | 1.03 | 1.05 | C(19)-S(20) | 1.75 | 1.66 |
| S(22)-O(30) | 1.46 | 1.45 | N(23)-C(24) | 1.40 | 1.46 | N(18)-H(39) | 1.03 | 1.05 |
| S(22)-O(29) | 1.46 | 1.45 | S(22)-O(26) | 1.46 | 1.45 | N(18)-C(19) | 1.38 | 1.46 |
| S(22)-N(23) | 1.72 | 1.71 | S(22)-O(25) | 1.46 | 1.45 | S(17)-O(25) | 1.46 | 1.45 |
| C(20)-C(21) | 1.38 | 1.42 | S(22)-N(23) | 1.70 | 1.71 | S(17)-O(24) | 1.46 | 1.45 |
| C(19)-S(22) | 1.76 | 1.71 | C(20)-C(21) | 1.38 | 1.42 | S(17)-N(18) | 1.74 | 1.71 |
| C(19)-C(20) | 1.40 | 1.42 | C(19)-S(22) | 1.76 | 1.71 | C(15)-C(16) | 1.39 | 1.42 |
| C(18)-C(19) | 1.39 | 1.42 | C(19)-C(20) | 1.40 | 1.42 | C(14)-S(17) | 1.76 | 1.71 |
| C(17)-C(18) | 1.39 | 1.42 | C(18)-C(19) | 1.40 | 1.42 | C(14)-C(15) | 1.40 | 1.42 |
| C(16)-C(21) | 1.40 | 1.42 | C(17)-C(18) | 1.39 | 1.42 | C(13)-C(14) | 1.39 | 1.42 |
| C(16)-C(17) | 1.40 | 1.42 | C(16)-C(21) | 1.40 | 1.42 | C(12)-C(13) | 1.39 | 1.42 |
| N(15)-H(41) | 1.06 | 1.05 | C(16)-C(17) | 1.40 | 1.42 | C(11)-C(16) | 1.40 | 1.42 |
| N(15)-C(16) | 1.38 | 1.46 | N(15)-H(42) | 1.07 | 1.05 | C(11)-C(12) | 1.40 | 1.42 |
| N(14)-N(15) | 1.31 | 1.43 | N(15)-C(16) | 1.38 | 1.46 | N(10)-H(34) | 1.04 | 1.05 |
| C(12)-O(32) | 1.24 | 1.21 | N(14)-N(15) | 1.31 | 1.43 | N(10)-C(11) | 1.38 | 1.46 |
| C(12)-O(31) | 1.34 | 1.34 | C(12)-O(33) | 1.24 | 1.21 | N(9)-N(10) | 1.31 | 1.43 |
| C(11)-C(12) | 1.44 | 1.52 | C(12)-O(32) | 1.34 | 1.34 | C(7)-O(31) | 1.23 | 1.21 |
| C(10)-N(14) | 1.31 | 1.26 | C(11)-C(12) | 1.44 | 1.52 | C(7)-O(30) | 1.37 | 1.34 |
| C(10)-C(11) | 1.46 | 1.50 | C(10)-N(14) | 1.31 | 1.26 | C(6)-C(7) | 1.44 | 1.52 |
| C(9)-O(13) | 1.22 | 1.21 | C(10)-C(11) | 1.46 | 1.50 | C(5)-N(9) | 1.31 | 1.26 |
| C(9)-C(10) | 1.48 | 1.52 | C(9)-O(13) | 1.22 | 1.21 | C(5)-C(6) | 1.46 | 1.50 |
| N(8)-H(40) | 1.02 | 1.05 | C(9)-C(10) | 1.48 | 1.52 | C(4)-O(8) | 1.22 | 1.21 |
| N(8)-C(9) | 1.40 | 1.46 | N(8)-H(41) | 1.02 | 1.05 | C(4)-C(5) | 1.48 | 1.52 |
| C(7)-C(11) | 1.40 | 1.34 | N(8)-C(9) | 1.40 | 1.46 | N(3)-H(33) | 1.02 | 1.05 |
| C(7)-N(8) | 1.37 | 1.46 | C(7)-C(11) | 1.40 | 1.34 | N(3)-C(4) | 1.39 | 1.46 |
| C(5)-C(6) | 1.39 | 1.42 | C(7)-N(8) | 1.37 | 1.46 | C(2)-C(6) | 1.41 | 1.34 |
| C(4)-C(5) | 1.39 | 1.42 | C(5)-C(6) | 1.39 | 1.42 | C(2)-N(3) | 1.37 | 1.46 |
| C(3)-C(7) | 1.46 | 1.50 | C(4)-C(5) | 1.39 | 1.42 | C(1)-S(29) | 1.74 | 1.66 |
| C(3)-C(4) | 1.40 | 1.42 | C(3)-C(7) | 1.46 | 1.50 | C(1)-C(26) | 1.40 | 1.42 |
| C(2)-C(3) | 1.40 | 1.42 | C(3)-C(4) | 1.40 | 1.42 | C(1)-C(2) | 1.43 | 1.50 |
| C(1)-C(6) | 1.40 | 1.42 | C(2)-C(3) | 1.40 | 1.42 | **RSMD** | **0.05** |  |
| C(1)-C(2) | 1.39 | 1.42 | C(1)-C(6) | 1.40 | 1.42 |  |  |  |
| **RSMD** | **0.05** |  | C(1)-C(2) | 1.39 | 1.42 |  |  |  |
|  |  |  | **RSMD** | **0.04** |  |  |  |  |
|  | **4f** | **Std** |  | **4b** | **Std** |  | **4e** | **Std** |
| O(31)-C(33) | 1.43 | 1.40 | O(32)-C(33) | 1.42 | 1.40 | O(33)-C(35) | 1.42 | 1.40 |
| C(29)-S(30) | 1.71 | 1.66 | C(27)-N(28) | 1.36 | 1.36 | C(30)-C(31) | 1.39 | 1.42 |
| C(28)-C(29) | 1.38 | 1.42 | C(26)-C(27) | 1.37 | 1.42 | C(29)-C(30) | 1.39 | 1.42 |
| C(27)-C(28) | 1.40 | 1.42 | S(25)-C(26) | 1.72 | 1.66 | N(28)-C(29) | 1.33 | 1.36 |
| C(25)-C(26) | 1.39 | 1.42 | C(24)-N(28) | 1.30 | 1.36 | C(27)-N(28) | 1.33 | 1.36 |
| C(24)-C(25) | 1.39 | 1.42 | C(24)-S(25) | 1.75 | 1.66 | C(24)-C(31) | 1.40 | 1.42 |
| N(23)-C(24) | 1.34 | 1.36 | N(23)-H(45) | 1.03 | 1.05 | C(24)-C(27) | 1.40 | 1.42 |
| C(22)-N(23) | 1.33 | 1.36 | N(23)-C(24) | 1.39 | 1.46 | N(23)-H(46) | 1.03 | 1.05 |
| C(19)-C(26) | 1.40 | 1.42 | S(22)-O(30) | 1.46 | 1.45 | N(23)-C(24) | 1.39 | 1.46 |
| C(19)-C(22) | 1.40 | 1.42 | S(22)-O(29) | 1.46 | 1.45 | S(22)-O(26) | 1.46 | 1.45 |
| N(18)-H(40) | 1.03 | 1.05 | S(22)-N(23) | 1.73 | 1.71 | S(22)-O(25) | 1.46 | 1.45 |
| N(18)-C(19) | 1.41 | 1.46 | C(20)-C(21) | 1.39 | 1.42 | S(22)-N(23) | 1.69 | 1.71 |
| S(17)-O(21) | 1.47 | 1.45 | C(19)-S(22) | 1.76 | 1.71 | C(20)-C(21) | 1.39 | 1.42 |
| S(17)-O(20) | 1.46 | 1.45 | C(19)-C(20) | 1.39 | 1.42 | C(19)-S(22) | 1.76 | 1.71 |
| S(17)-N(18) | 1.71 | 1.71 | C(18)-C(19) | 1.39 | 1.42 | C(19)-C(20) | 1.39 | 1.42 |
| C(15)-C(16) | 1.38 | 1.42 | C(17)-C(18) | 1.39 | 1.42 | C(18)-C(19) | 1.39 | 1.42 |
| C(14)-S(17) | 1.76 | 1.71 | C(16)-C(21) | 1.40 | 1.42 | C(17)-C(18) | 1.38 | 1.42 |
| C(14)-C(15) | 1.40 | 1.42 | C(16)-C(17) | 1.40 | 1.42 | C(16)-C(21) | 1.40 | 1.42 |
| C(13)-C(14) | 1.39 | 1.42 | N(15)-H(40) | 1.06 | 1.05 | C(16)-C(17) | 1.40 | 1.42 |
| C(12)-C(13) | 1.39 | 1.42 | N(15)-C(16) | 1.38 | 1.46 | N(15)-H(41) | 1.06 | 1.05 |
| C(11)-C(16) | 1.40 | 1.42 | N(14)-N(15) | 1.31 | 1.43 | N(15)-C(16) | 1.38 | 1.46 |
| C(11)-C(12) | 1.40 | 1.42 | C(12)-O(34) | 1.24 | 1.21 | N(14)-N(15) | 1.31 | 1.43 |
| N(10)-H(35) | 1.04 | 1.05 | C(12)-O(32) | 1.34 | 1.34 | C(12)-O(34) | 1.24 | 1.21 |
| N(10)-C(11) | 1.38 | 1.46 | C(11)-C(12) | 1.44 | 1.52 | C(12)-O(33) | 1.34 | 1.34 |
| N(9)-N(10) | 1.31 | 1.43 | C(10)-N(14) | 1.30 | 1.26 | C(11)-C(12) | 1.44 | 1.52 |
| C(7)-O(32) | 1.23 | 1.21 | C(10)-C(11) | 1.45 | 1.50 | C(10)-N(14) | 1.31 | 1.26 |
| C(7)-O(31) | 1.37 | 1.34 | C(9)-O(13) | 1.22 | 1.21 | C(10)-C(11) | 1.46 | 1.50 |
| C(6)-C(7) | 1.43 | 1.52 | C(9)-C(10) | 1.48 | 1.52 | C(9)-O(13) | 1.22 | 1.21 |
| C(5)-N(9) | 1.31 | 1.26 | N(8)-H(39) | 1.02 | 1.05 | C(9)-C(10) | 1.48 | 1.52 |
| C(5)-C(6) | 1.46 | 1.50 | N(8)-C(9) | 1.40 | 1.46 | N(8)-H(40) | 1.02 | 1.05 |
| C(4)-O(8) | 1.22 | 1.21 | C(7)-C(11) | 1.40 | 1.34 | N(8)-C(9) | 1.40 | 1.46 |
| C(4)-C(5) | 1.48 | 1.52 | C(7)-N(8) | 1.37 | 1.46 | C(7)-C(11) | 1.40 | 1.34 |
| N(3)-H(34) | 1.02 | 1.05 | C(6)-Br(31) | 1.88 | 1.88 | C(7)-N(8) | 1.37 | 1.46 |
| N(3)-C(4) | 1.39 | 1.46 | C(5)-C(6) | 1.39 | 1.42 | C(6)-Br(32) | 1.88 | 1.88 |
| C(2)-C(6) | 1.41 | 1.34 | C(4)-C(5) | 1.39 | 1.42 | C(5)-C(6) | 1.39 | 1.42 |
| C(2)-N(3) | 1.37 | 1.46 | C(3)-C(7) | 1.46 | 1.50 | C(4)-C(5) | 1.39 | 1.42 |
| C(1)-S(30) | 1.74 | 1.66 | C(3)-C(4) | 1.40 | 1.42 | C(3)-C(7) | 1.45 | 1.50 |
| C(1)-C(27) | 1.40 | 1.42 | C(2)-C(3) | 1.40 | 1.42 | C(3)-C(4) | 1.40 | 1.42 |
| C(1)-C(2) | 1.43 | 1.50 | C(1)-C(6) | 1.39 | 1.42 | C(2)-C(3) | 1.40 | 1.42 |
| **RSMD** | **0.05** |  | C(1)-C(2) | 1.39 | 1.42 | C(1)-C(6) | 1.39 | 1.42 |
|  |  |  | **RSMD** | **0.05** |  | C(1)-C(2) | 1.39 | 1.42 |
|  |  |  |  |  |  | **RSMD** | **0.04** |  |

**Table S3.** The DFT bond angle data of the investigated compounds.

|  | **4a** |  |  | **4d** |  |  | **4c** |  |
| --- | --- | --- | --- | --- | --- | --- | --- | --- |
| C(34)-C(33)-O(31) | 111.1 | 107.4 | C(35)-C(34)-O(32) | 111.1 | 107.4 | C(32)-O(30)-C(7) | 114.0 | 109.9 |
| C(33)-O(31)-C(12) | 116.0 | 109.9 | C(34)-O(32)-C(12) | 116.0 | 109.9 | C(28)-S(29)-C(1) | 91.6 | 98.5 |
| C(27)-N(28)-C(24) | 110.6 | 115.0 | C(30)-C(31)-C(24) | 118.8 | 120.0 | S(29)-C(28)-C(27) | 113.1 | 109.0 |
| N(28)-C(27)-C(26) | 116.2 | 123.5 | C(31)-C(30)-C(29) | 118.7 | 120.0 | C(28)-C(27)-C(26) | 111.5 | 109.0 |
| C(27)-C(26)-S(25) | 109.7 | 107.0 | C(30)-C(29)-N(28) | 123.0 | 123.5 | C(27)-C(26)-C(1) | 113.8 | 109.0 |
| C(26)-S(25)-C(24) | 88.8 | 98.5 | C(29)-N(28)-C(27) | 118.4 | 115.0 | C(22)-N(23)-C(19) | 110.7 | 115.0 |
| N(28)-C(24)-S(25) | 114.7 | 126.0 | N(28)-C(27)-C(24) | 123.0 | 123.5 | N(23)-C(22)-C(21) | 116.2 | 123.5 |
| N(28)-C(24)-N(23) | 124.0 | 126.0 | C(31)-C(24)-C(27) | 118.1 | 120.0 | C(22)-C(21)-S(20) | 109.7 | 109.0 |
| S(25)-C(24)-N(23) | 121.3 | 120.0 | C(31)-C(24)-N(23) | 120.5 | 120.0 | C(21)-S(20)-C(19) | 88.6 | 98.5 |
| H(46)-N(23)-C(24) | 119.5 | 118.0 | C(27)-C(24)-N(23) | 121.3 | 120.0 | N(23)-C(19)-S(20) | 114.8 | 126.0 |
| H(46)-N(23)-S(22) | 110.7 | 109.0 | H(47)-N(23)-C(24) | 115.4 | 118.0 | N(23)-C(19)-N(18) | 123.8 | 126.0 |
| C(24)-N(23)-S(22) | 122.1 | 120.0 | H(47)-N(23)-S(22) | 109.4 | 107.0 | S(20)-C(19)-N(18) | 121.5 | 120.0 |
| O(30)-S(22)-O(29) | 123.3 | 116.6 | C(24)-N(23)-S(22) | 120.7 | 120.0 | H(39)-N(18)-C(19) | 115.4 | 118.0 |
| O(30)-S(22)-N(23) | 108.4 | 107.0 | O(26)-S(22)-O(25) | 123.7 | 116.6 | H(39)-N(18)-S(17) | 110.2 | 109.0 |
| O(30)-S(22)-C(19) | 109.1 | 107.0 | O(26)-S(22)-N(23) | 103.2 | 107.0 | C(19)-N(18)-S(17) | 116.7 | 120.0 |
| O(29)-S(22)-N(23) | 101.5 | 107.0 | O(26)-S(22)-C(19) | 109.2 | 107.0 | O(25)-S(17)-O(24) | 122.0 | 116.6 |
| O(29)-S(22)-C(19) | 108.7 | 107.0 | O(25)-S(22)-N(23) | 106.4 | 107.0 | O(25)-S(17)-N(18) | 107.4 | 107.0 |
| N(23)-S(22)-C(19) | 104.0 | 107.0 | O(25)-S(22)-C(19) | 108.0 | 107.0 | O(25)-S(17)-C(14) | 109.5 | 107.0 |
| C(20)-C(21)-C(16) | 119.2 | 120.0 | N(23)-S(22)-C(19) | 104.6 | 107.0 | O(24)-S(17)-N(18) | 108.8 | 107.0 |
| C(21)-C(20)-C(19) | 119.3 | 120.0 | C(20)-C(21)-C(16) | 119.3 | 120.0 | O(24)-S(17)-C(14) | 109.1 | 107.0 |
| S(22)-C(19)-C(20) | 118.6 | 120.0 | C(21)-C(20)-C(19) | 119.5 | 120.0 | N(18)-S(17)-C(14) | 97.1 | 98.0 |
| S(22)-C(19)-C(18) | 119.3 | 120.0 | S(22)-C(19)-C(20) | 119.1 | 120.0 | C(15)-C(16)-C(11) | 119.2 | 120.0 |
| C(20)-C(19)-C(18) | 122.1 | 120.0 | S(22)-C(19)-C(18) | 119.3 | 120.0 | C(16)-C(15)-C(14) | 119.5 | 120.0 |
| C(19)-C(18)-C(17) | 118.5 | 120.0 | C(20)-C(19)-C(18) | 121.6 | 120.0 | S(17)-C(14)-C(15) | 119.4 | 120.0 |
| C(18)-C(17)-C(16) | 120.0 | 120.0 | C(19)-C(18)-C(17) | 118.9 | 120.0 | S(17)-C(14)-C(13) | 118.8 | 120.0 |
| C(21)-C(16)-C(17) | 120.8 | 120.0 | C(18)-C(17)-C(16) | 119.9 | 120.0 | C(15)-C(14)-C(13) | 121.8 | 120.0 |
| C(21)-C(16)-N(15) | 121.3 | 120.0 | C(21)-C(16)-C(17) | 120.7 | 120.0 | C(14)-C(13)-C(12) | 118.8 | 120.0 |
| C(17)-C(16)-N(15) | 117.9 | 120.0 | C(21)-C(16)-N(15) | 121.2 | 120.0 | C(13)-C(12)-C(11) | 119.9 | 120.0 |
| H(41)-N(15)-C(16) | 121.3 | 118.0 | C(17)-C(16)-N(15) | 118.1 | 120.0 | C(16)-C(11)-C(12) | 120.7 | 120.0 |
| H(41)-N(15)-N(14) | 118.7 | 113.0 | H(42)-N(15)-C(16) | 121.4 | 118.0 | C(16)-C(11)-N(10) | 120.9 | 120.0 |
| C(16)-N(15)-N(14) | 120.1 | 124.0 | H(42)-N(15)-N(14) | 118.6 | 113.0 | C(12)-C(11)-N(10) | 118.3 | 120.0 |
| N(15)-N(14)-C(10) | 121.1 | 115.0 | C(16)-N(15)-N(14) | 119.9 | 124.0 | H(34)-N(10)-C(11) | 120.4 | 118.0 |
| O(32)-C(12)-O(31) | 120.5 | 122.0 | N(15)-N(14)-C(10) | 121.1 | 115.0 | H(34)-N(10)-N(9) | 119.5 | 113.0 |
| O(32)-C(12)-C(11) | 125.2 | 123.0 | O(33)-C(12)-O(32) | 120.5 | 122.0 | C(11)-N(10)-N(9) | 120.1 | 124.0 |
| O(31)-C(12)-C(11) | 114.3 | 124.3 | O(33)-C(12)-C(11) | 125.1 | 123.0 | N(10)-N(9)-C(5) | 122.7 | 115.0 |
| C(12)-C(11)-C(10) | 127.0 | 117.6 | O(32)-C(12)-C(11) | 114.3 | 124.3 | O(31)-C(7)-O(30) | 119.2 | 122.0 |
| C(12)-C(11)-C(7) | 125.9 | 117.6 | C(12)-C(11)-C(10) | 126.9 | 117.6 | O(31)-C(7)-C(6) | 125.7 | 123.0 |
| C(10)-C(11)-C(7) | 106.7 | 120.0 | C(12)-C(11)-C(7) | 126.0 | 117.6 | O(30)-C(7)-C(6) | 115.1 | 124.3 |
| N(14)-C(10)-C(11) | 136.3 | 120.0 | C(10)-C(11)-C(7) | 106.7 | 120.0 | C(7)-C(6)-C(5) | 131.8 | 117.6 |
| N(14)-C(10)-C(9) | 116.3 | 120.0 | N(14)-C(10)-C(11) | 136.4 | 120.0 | C(7)-C(6)-C(2) | 121.2 | 117.6 |
| C(11)-C(10)-C(9) | 107.4 | 117.6 | N(14)-C(10)-C(9) | 116.2 | 120.0 | C(5)-C(6)-C(2) | 107.0 | 120.0 |
| O(13)-C(9)-C(10) | 131.5 | 123.0 | C(11)-C(10)-C(9) | 107.4 | 117.6 | N(9)-C(5)-C(6) | 138.4 | 120.0 |
| O(13)-C(9)-N(8) | 124.5 | 122.6 | O(13)-C(9)-C(10) | 131.5 | 123.0 | N(9)-C(5)-C(4) | 114.6 | 120.0 |
| C(10)-C(9)-N(8) | 103.9 | 122.0 | O(13)-C(9)-N(8) | 124.6 | 122.6 | C(6)-C(5)-C(4) | 107.1 | 117.6 |
| H(40)-N(8)-C(9) | 122.1 | 118.0 | C(10)-C(9)-N(8) | 103.9 | 122.0 | O(8)-C(4)-C(5) | 130.8 | 123.0 |
| H(40)-N(8)-C(7) | 124.1 | 118.0 | H(41)-N(8)-C(9) | 122.1 | 118.0 | O(8)-C(4)-N(3) | 124.8 | 122.6 |
| C(9)-N(8)-C(7) | 112.6 | 109.0 | H(41)-N(8)-C(7) | 124.1 | 118.0 | C(5)-C(4)-N(3) | 104.3 | 122.0 |
| C(11)-C(7)-N(8) | 109.3 | 120.0 | C(9)-N(8)-C(7) | 112.6 | 109.0 | H(33)-N(3)-C(4) | 121.3 | 118.0 |
| C(11)-C(7)-C(3) | 133.2 | 120.0 | C(11)-C(7)-N(8) | 109.3 | 120.0 | H(33)-N(3)-C(2) | 124.8 | 118.0 |
| N(8)-C(7)-C(3) | 117.5 | 120.0 | C(11)-C(7)-C(3) | 133.2 | 120.0 | C(4)-N(3)-C(2) | 113.0 | 109.0 |
| C(5)-C(6)-C(1) | 119.8 | 120.0 | N(8)-C(7)-C(3) | 117.5 | 120.0 | C(6)-C(2)-N(3) | 108.5 | 120.0 |
| C(6)-C(5)-C(4) | 120.0 | 120.0 | C(5)-C(6)-C(1) | 119.8 | 120.0 | C(6)-C(2)-C(1) | 134.8 | 120.0 |
| C(5)-C(4)-C(3) | 120.5 | 120.0 | C(6)-C(5)-C(4) | 120.0 | 120.0 | N(3)-C(2)-C(1) | 116.6 | 120.0 |
| C(7)-C(3)-C(4) | 118.9 | 120.0 | C(5)-C(4)-C(3) | 120.5 | 120.0 | S(29)-C(1)-C(26) | 109.9 | 119.0 |
| C(7)-C(3)-C(2) | 121.9 | 120.0 | C(7)-C(3)-C(4) | 118.8 | 120.0 | S(29)-C(1)-C(2) | 127.7 | 119.0 |
| C(4)-C(3)-C(2) | 119.1 | 120.0 | C(7)-C(3)-C(2) | 122.0 | 120.0 | C(26)-C(1)-C(2) | 122.3 | 120.0 |
| C(3)-C(2)-C(1) | 120.2 | 120.0 | C(4)-C(3)-C(2) | 119.1 | 120.0 | **RSMD** | **6.7** |  |
| C(6)-C(1)-C(2) | 120.4 | 120.0 | C(3)-C(2)-C(1) | 120.2 | 120.0 |  |  |  |
| **RSMD** | **6.1** |  | C(6)-C(1)-C(2) | 120.4 | 120.0 |  |  |  |
|  |  |  | **RSMD** | **5.6** |  |  |  |  |
|  | **4f** |  |  | **4b** |  |  | **4e** |  |
| C(33)-O(31)-C(7) | 114.0 | 109.9 | C(33)-O(32)-C(12) | 115.1 | 109.9 | C(35)-O(33)-C(12) | 114.8 | 109.9 |
| C(29)-S(30)-C(1) | 91.7 | 98.5 | C(27)-N(28)-C(24) | 110.6 | 115.0 | C(30)-C(31)-C(24) | 118.9 | 120.0 |
| S(30)-C(29)-C(28) | 113.1 | 109.9 | N(28)-C(27)-C(26) | 116.2 | 123.5 | C(31)-C(30)-C(29) | 118.8 | 120.0 |
| C(29)-C(28)-C(27) | 111.5 | 109.9 | C(27)-C(26)-S(25) | 109.7 | 109.9 | C(30)-C(29)-N(28) | 122.7 | 123.5 |
| C(28)-C(27)-C(1) | 113.8 | 109.9 | C(26)-S(25)-C(24) | 88.5 | 98.5 | C(29)-N(28)-C(27) | 118.7 | 115.0 |
| C(25)-C(26)-C(19) | 118.7 | 120.0 | N(28)-C(24)-S(25) | 114.9 | 126.0 | N(28)-C(27)-C(24) | 123.1 | 123.5 |
| C(26)-C(25)-C(24) | 118.6 | 120.0 | N(28)-C(24)-N(23) | 124.7 | 126.0 | C(31)-C(24)-C(27) | 117.8 | 120.0 |
| C(25)-C(24)-N(23) | 123.1 | 123.5 | S(25)-C(24)-N(23) | 120.4 | 120.0 | C(31)-C(24)-N(23) | 119.1 | 120.0 |
| C(24)-N(23)-C(22) | 118.3 | 115.0 | H(45)-N(23)-C(24) | 115.2 | 118.0 | C(27)-C(24)-N(23) | 123.1 | 120.0 |
| N(23)-C(22)-C(19) | 123.0 | 123.5 | H(45)-N(23)-S(22) | 110.3 | 109.0 | H(46)-N(23)-C(24) | 118.3 | 118.0 |
| C(26)-C(19)-C(22) | 118.2 | 120.0 | C(24)-N(23)-S(22) | 118.5 | 120.0 | H(46)-N(23)-S(22) | 110.2 | 109.0 |
| C(26)-C(19)-N(18) | 120.6 | 120.0 | O(30)-S(22)-O(29) | 121.9 | 116.6 | C(24)-N(23)-S(22) | 126.7 | 120.0 |
| C(22)-C(19)-N(18) | 121.1 | 120.0 | O(30)-S(22)-N(23) | 109.9 | 109.0 | O(26)-S(22)-O(25) | 123.0 | 116.6 |
| H(40)-N(18)-C(19) | 112.9 | 118.0 | O(30)-S(22)-C(19) | 108.4 | 109.0 | O(26)-S(22)-N(23) | 110.0 | 109.0 |
| H(40)-N(18)-S(17) | 108.2 | 107.0 | O(29)-S(22)-N(23) | 106.9 | 109.0 | O(26)-S(22)-C(19) | 107.4 | 109.0 |
| C(19)-N(18)-S(17) | 119.6 | 120.0 | O(29)-S(22)-C(19) | 110.4 | 109.0 | O(25)-S(22)-N(23) | 101.6 | 98.0 |
| O(21)-S(17)-O(20) | 121.7 | 116.6 | N(23)-S(22)-C(19) | 96.3 | 98.0 | O(25)-S(22)-C(19) | 109.5 | 109.0 |
| O(21)-S(17)-N(18) | 109.6 | 109.0 | C(20)-C(21)-C(16) | 119.3 | 120.0 | N(23)-S(22)-C(19) | 103.7 | 98.0 |
| O(21)-S(17)-C(14) | 107.3 | 109.0 | C(21)-C(20)-C(19) | 119.4 | 120.0 | C(20)-C(21)-C(16) | 119.3 | 120.0 |
| O(20)-S(17)-N(18) | 105.5 | 109.0 | S(22)-C(19)-C(20) | 119.1 | 120.0 | C(21)-C(20)-C(19) | 119.6 | 120.0 |
| O(20)-S(17)-C(14) | 110.5 | 109.0 | S(22)-C(19)-C(18) | 119.0 | 120.0 | S(22)-C(19)-C(20) | 119.4 | 120.0 |
| N(18)-S(17)-C(14) | 100.2 | 98.0 | C(20)-C(19)-C(18) | 121.8 | 120.0 | S(22)-C(19)-C(18) | 119.0 | 120.0 |
| C(15)-C(16)-C(11) | 119.3 | 120.0 | C(19)-C(18)-C(17) | 118.9 | 120.0 | C(20)-C(19)-C(18) | 121.6 | 120.0 |
| C(16)-C(15)-C(14) | 119.6 | 120.0 | C(18)-C(17)-C(16) | 119.7 | 120.0 | C(19)-C(18)-C(17) | 119.1 | 120.0 |
| S(17)-C(14)-C(15) | 119.7 | 120.0 | C(21)-C(16)-C(17) | 120.9 | 120.0 | C(18)-C(17)-C(16) | 119.8 | 120.0 |
| S(17)-C(14)-C(13) | 118.7 | 120.0 | C(21)-C(16)-N(15) | 121.1 | 120.0 | C(21)-C(16)-C(17) | 120.8 | 120.0 |
| C(15)-C(14)-C(13) | 121.6 | 120.0 | C(17)-C(16)-N(15) | 118.0 | 120.0 | C(21)-C(16)-N(15) | 121.4 | 120.0 |
| C(14)-C(13)-C(12) | 119.0 | 120.0 | H(40)-N(15)-C(16) | 121.1 | 118.0 | C(17)-C(16)-N(15) | 117.9 | 120.0 |
| C(13)-C(12)-C(11) | 119.9 | 120.0 | H(40)-N(15)-N(14) | 118.6 | 113.0 | H(41)-N(15)-C(16) | 121.3 | 118.0 |
| C(16)-C(11)-C(12) | 120.7 | 120.0 | C(16)-N(15)-N(14) | 120.1 | 124.0 | H(41)-N(15)-N(14) | 118.7 | 113.0 |
| C(16)-C(11)-N(10) | 120.9 | 120.0 | N(15)-N(14)-C(10) | 119.5 | 115.0 | C(16)-N(15)-N(14) | 120.0 | 124.0 |
| C(12)-C(11)-N(10) | 118.5 | 120.0 | O(34)-C(12)-O(32) | 120.6 | 122.0 | N(15)-N(14)-C(10) | 121.2 | 115.0 |
| H(35)-N(10)-C(11) | 120.3 | 118.0 | O(34)-C(12)-C(11) | 124.5 | 123.0 | O(34)-C(12)-O(33) | 119.9 | 122.0 |
| H(35)-N(10)-N(9) | 119.5 | 113.0 | O(32)-C(12)-C(11) | 114.9 | 124.3 | O(34)-C(12)-C(11) | 125.5 | 123.0 |
| C(11)-N(10)-N(9) | 120.1 | 124.0 | C(12)-C(11)-C(10) | 125.3 | 117.6 | O(33)-C(12)-C(11) | 114.6 | 124.3 |
| N(10)-N(9)-C(5) | 122.8 | 115.0 | C(12)-C(11)-C(7) | 127.5 | 117.6 | C(12)-C(11)-C(10) | 126.8 | 117.6 |
| O(32)-C(7)-O(31) | 119.2 | 122.0 | C(10)-C(11)-C(7) | 106.8 | 120.0 | C(12)-C(11)-C(7) | 126.1 | 117.6 |
| O(32)-C(7)-C(6) | 125.7 | 123.0 | N(14)-C(10)-C(11) | 135.1 | 120.0 | C(10)-C(11)-C(7) | 106.7 | 120.0 |
| O(31)-C(7)-C(6) | 115.1 | 124.3 | N(14)-C(10)-C(9) | 117.2 | 120.0 | N(14)-C(10)-C(11) | 136.4 | 120.0 |
| C(7)-C(6)-C(5) | 131.8 | 117.6 | C(11)-C(10)-C(9) | 107.8 | 117.6 | N(14)-C(10)-C(9) | 116.2 | 120.0 |
| C(7)-C(6)-C(2) | 121.2 | 117.6 | O(13)-C(9)-C(10) | 131.5 | 123.0 | C(11)-C(10)-C(9) | 107.4 | 117.6 |
| C(5)-C(6)-C(2) | 107.0 | 120.0 | O(13)-C(9)-N(8) | 124.8 | 122.6 | O(13)-C(9)-C(10) | 131.5 | 123.0 |
| N(9)-C(5)-C(6) | 138.3 | 120.0 | C(10)-C(9)-N(8) | 103.7 | 122.0 | O(13)-C(9)-N(8) | 124.5 | 122.6 |
| N(9)-C(5)-C(4) | 114.6 | 120.0 | H(39)-N(8)-C(9) | 122.4 | 118.0 | C(10)-C(9)-N(8) | 104.0 | 122.0 |
| C(6)-C(5)-C(4) | 107.1 | 117.6 | H(39)-N(8)-C(7) | 124.5 | 118.0 | H(40)-N(8)-C(9) | 121.9 | 118.0 |
| O(8)-C(4)-C(5) | 130.9 | 123.0 | C(9)-N(8)-C(7) | 112.6 | 109.0 | H(40)-N(8)-C(7) | 124.3 | 118.0 |
| O(8)-C(4)-N(3) | 124.8 | 122.6 | C(11)-C(7)-N(8) | 109.1 | 120.0 | C(9)-N(8)-C(7) | 112.6 | 109.0 |
| C(5)-C(4)-N(3) | 104.3 | 122.0 | C(11)-C(7)-C(3) | 133.5 | 120.0 | C(11)-C(7)-N(8) | 109.3 | 120.0 |
| H(34)-N(3)-C(4) | 121.4 | 118.0 | N(8)-C(7)-C(3) | 117.3 | 120.0 | C(11)-C(7)-C(3) | 133.1 | 120.0 |
| H(34)-N(3)-C(2) | 124.8 | 118.0 | Br(31)-C(6)-C(5) | 119.5 | 118.1 | N(8)-C(7)-C(3) | 117.6 | 120.0 |
| C(4)-N(3)-C(2) | 113.0 | 109.0 | Br(31)-C(6)-C(1) | 119.4 | 118.1 | Br(32)-C(6)-C(5) | 119.5 | 118.1 |
| C(6)-C(2)-N(3) | 108.5 | 120.0 | C(5)-C(6)-C(1) | 121.1 | 120.0 | Br(32)-C(6)-C(1) | 119.4 | 118.1 |
| C(6)-C(2)-C(1) | 134.8 | 120.0 | C(6)-C(5)-C(4) | 119.0 | 120.0 | C(5)-C(6)-C(1) | 121.1 | 120.0 |
| N(3)-C(2)-C(1) | 116.6 | 120.0 | C(5)-C(4)-C(3) | 121.0 | 120.0 | C(6)-C(5)-C(4) | 119.0 | 120.0 |
| S(30)-C(1)-C(27) | 109.9 | 119.0 | C(7)-C(3)-C(4) | 118.7 | 120.0 | C(5)-C(4)-C(3) | 121.0 | 120.0 |
| S(30)-C(1)-C(2) | 127.7 | 119.0 | C(7)-C(3)-C(2) | 122.3 | 120.0 | C(7)-C(3)-C(4) | 119.0 | 120.0 |
| C(27)-C(1)-C(2) | 122.3 | 120.0 | C(4)-C(3)-C(2) | 118.8 | 120.0 | C(7)-C(3)-C(2) | 122.1 | 120.0 |
| **RSMD** | **6.3** |  | C(3)-C(2)-C(1) | 120.7 | 120.0 | C(4)-C(3)-C(2) | 118.8 | 120.0 |
|  |  |  | C(6)-C(1)-C(2) | 119.4 | 120.0 | C(3)-C(2)-C(1) | 120.8 | 120.0 |
|  |  |  | **RSMD** | **5.9** |  | C(6)-C(1)-C(2) | 119.4 | 120.0 |
|  |  |  |  |  |  | **RSMD** | **5.6** |  |

**Table S4.** The results of minimal inhibition (MIC µg/mL) and the zone diameter (IZD mm) of the synthesized sulphonamide hybrids.

| Hybrids | Gram-positive bacteria | | Gram- negative bacteria | |
| --- | --- | --- | --- | --- |
|  | *S. aureus* | *B. subtilis* | *S. typhimurium* | *E.coli* |
| ***4 a*** | 21 (11.31) | 17(11.31) | 15(19.24) | 19(11.31) |
| ***4b*** | 18(18.52) | 16(11.31) | 23(11.31) | 20(11.31) |
| ***4c*** | 23(11.31) | 25(11.31) | 19(19.24) | 25(11.31) |
| ***4d*** | 26 (11.31) | 18(11.31) | 16(19.24) | 21(11.31) |
| ***4e*** | 16(19.24) | 14(21.43) | NA | 16(19.24) |
| ***4f*** | 20(11.31) | 21(11.31) | 20(19.24) | 18(11.31) |
| Sulfamethoxazole | 16(11.31) | 16(11.31) | 20(11.31) | 19(11.31) |

Notes: (IZD) inhibition zone diameter (mm), (MIC) minimum inhibition concentration (µg/mL), (NA) no activity,

and Sulfamethoxazole is a standard antibiotic.

**Table S5.** Molecular docking data of **sulphonamide** -based analogues against PDB:3TZF.

| **Code** | **S(Kcal/mol)** | **Rmsd** | **ligand Interactions** | **Types of bindings** | **Distance (Å)** |
| --- | --- | --- | --- | --- | --- |
| **Hybrid 4a** | ‐7.7808 | 1.5695 | N 8 with Asp 96  N 22 with Ser 222  O 29 with Ser 222  O 31 with Thr 62  6- ring with Arg 63 | H-donor  H-donor  H-acceptor  H-acceptor  π-H | 2.84  2.76  2.68  2.82  4.37 |
| **Hybrid 4b** | ‐7.7017 | 1.5621 | N 22 with Asn 22  O 24 with Ser 27  O 24 with Ser 61  O 25 with Asn 22 | H-donor  H-acceptor  H-acceptor  H-acceptor | 2.88  2.94  3.34  2.73 |
| **Hybrid 4c** | ‐8.0956 | 1.2875 | N 3 with Asp 96  N 8 with Thr 62  O 30 with Arg 239 | H-donor  H-acceptor  H-acceptor | 3.28  3.31  2.81 |
| **Hybrid 4d** | ‐7.5402 | 1.6077 | N 22 with Asp 96  S 24 with Asp 96  N 27 with Lys 221  O 28 with Thr 62  O 29 with Arg 255 | H-donor  H-donor  H-acceptor  H-acceptor  H-acceptor | 3.56  3.46  3.40  2.88  3.05 |
| **Hybrid 4e** | ‐7.6154 | 1.4262 | O 24 with Thr 62  O 25 with Arg 255  N 27 with Lys 221  6-ring with Phe 190 | H-acceptor  H-acceptor  H-acceptor  π- H | 3.47  3.31  3.33  4.04 |
| **Hybrid 4f** | ‐7.6833 | 1.1397 | N 3 with Asp 96  O 20 with Ser 222  C 25 with Phe 28  5-ring with Ser 61  6-ring with Arg 63 | H-donor  H-acceptor  H-acceptor  H-π  π- H | 2.76  3.49  2.67  4.63  4.52 |

**Table S6.** Molecular docking data of **sulphonamide** -based analogues against PDB: 6CLV.

| **Code** | **S(Kcal/mol)** | **Rmsd** | **ligand Interactions** | **Types of bindings** | **Distance (Å)** |
| --- | --- | --- | --- | --- | --- |
| **Hybrid 4a** | -7.2950 | 1.4467 | N 8 with Val 49  O 12 with Arg 52  O 28 with Arg 204  O 31 with Arg 239  5- ring with Arg 52  6- ring with Lys 203 | H-donor  H-acceptor  H-acceptor  H-acceptor  π-cation  π-H | 3.20  3.16  2.85  3.33  4.06  4.24 |
| **Hybrid 4b** | -7.9159 | 1.4346 | N 8 with Gly 48  O 24 with Arg 239  O 25 with Lys 203 | H-donor  H-acceptor  H-acceptor | 3.03  2.76  3.28 |
| **Hybrid 4c** | -7.3906 | 1.4172 | N 3 with Asp 84  O 7 with Met 128  S 28 with Asp 84  N 22 with Arg 176  O 23 with Arg 204  O 30 with Arg 239  6-ring with Arg 53  6-ring with Lus 203 | H-donor  H-donor  H-donor  H-acceptor  H-acceptor  H-acceptor  π-H  π- H | 2.96  4.24  3.37  3.24  3.43  2.99  3.97  4.07 |
| **Hybrid 4d** | -7.1042 | 1.4640 | S 24 with Asp 15  O 29 with Arg 239  5-ring with Ser 50  6-ring with Arg 176  5- ring with Arg 204 | H-donor  H-acceptor  π-H  π-cation  π-H | 3.98  3.23  4.83  3.95  4.27 |
| **Hybrid 4e** | -8.0112 | 1.4210 | N 8 with Val 49  C 17 with Gly 171  O 25 with Arg 204  O 33 with Arg 239  6-ring with Ser 50 | H-donor  H-donor  H-acceptor  H-acceptor  π- H | 3.10  3.26  2.97  3.26  4.28 |
| **Hybrid 4f** | -7.7906 | 0.9648 | N 3 with Asp 84  S 29 with Asp 84  6-ring with Ser 50 | H-donor  H-donor  π-H | 2.70  3.43  4.05 |

**Table S7.** Physicochemical parameter values for Sulphonamide hybrids using Swiss ADME software.

| **Hybrids** | MW ^a^ | **nHA ^b^** | **nAHA ^c^** | **nRotB ^d^** | nHBA ^e^ | **nHBD ^f^** | **MR ^g^** | **TPSA ^h^** | **MLOGP ^i^** | **ESOL ^j^** |
| --- | --- | --- | --- | --- | --- | --- | --- | --- | --- | --- |
| 4a | 497.55 | 34 | 17 | 9 | 7 | 3 | 131.95 | 175.47 | 0.83 | MS |
| 4b | 562.42 | 34 | 17 | 8 | 7 | 3 | 134.85 | 175.47 | 1.20 | MS |
| 4c | 489.55 | 32 | 16 | 8 | 7 | 3 | 125.02 | 203.71 | 0.19 | MS |
| 4d | 491.52 | 35 | 18 | 9 | 7 | 3 | 134.08 | 147.23 | 0.81 | MS |
| 4e | 556.39 | 35 | 18 | 8 | 7 | 3 | 136.97 | 147.23 | 1.19 | MS |
| 4f | 483.52 | 33 | 17 | 8 | 7 | 3 | 127.15 | 175.47 | 0.20 | MS |

^a^ Molecular weight (MW), ^b^ number of heavy atoms (nHA), ^c^ number of aromatic heavy atoms (nAH^A^), ^d^ number

of rotatable bonds (nRot^B^), e number of hydrogen bond acceptors (nHB^A^), f number of hydrogen bond donors

(nHBD), ^g^ molecular refractivity (MR), ^h^ topological polar surface area (TPSA), ^i^ octanol/water partition coefficient

(MLOGP), and ^j^ ESOL (estimated SOLubility) with MS moderately soluble and S representing soluble.

**Spectral Charts**

**
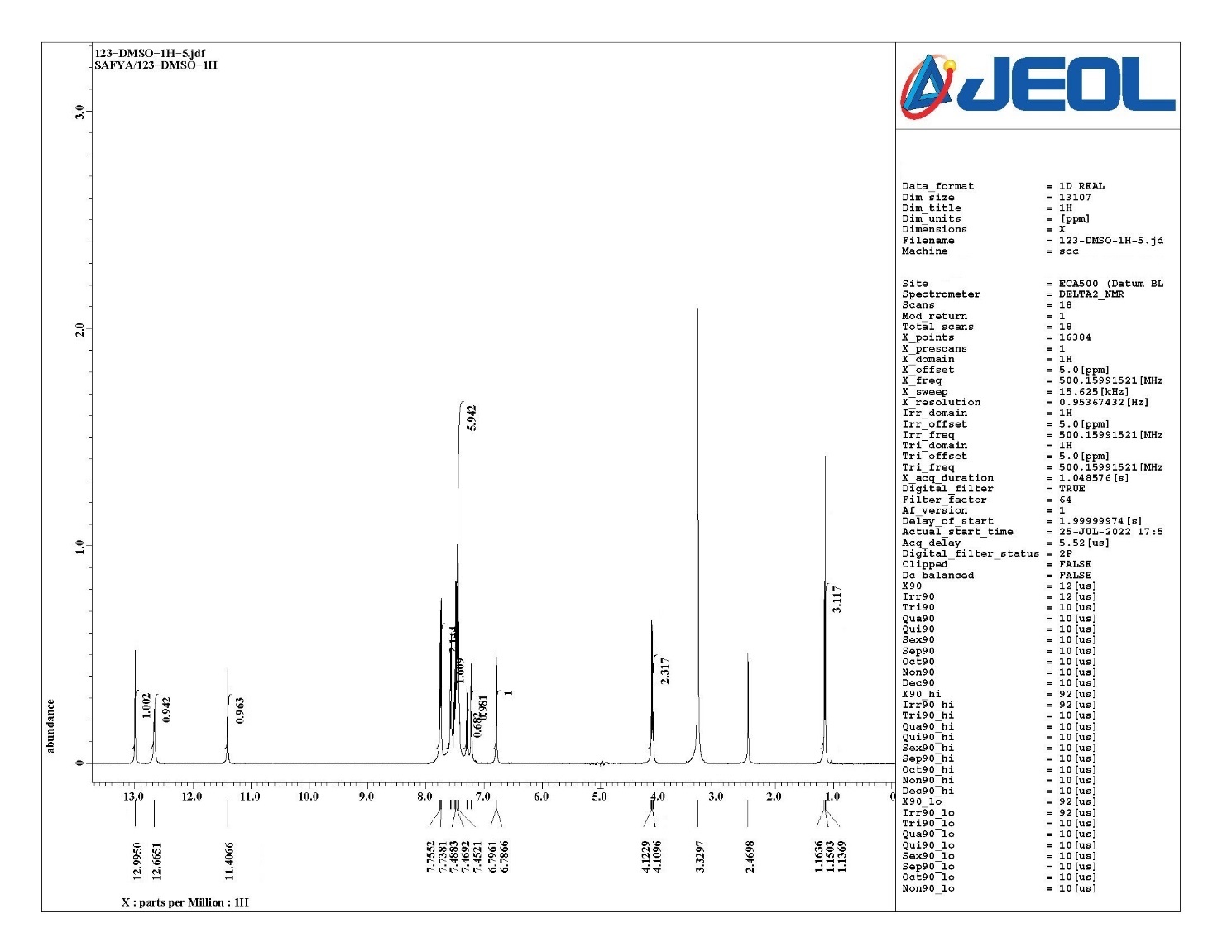
**

**Figure S1: ^1^H-NMR spectrum of compound 4a.**

**
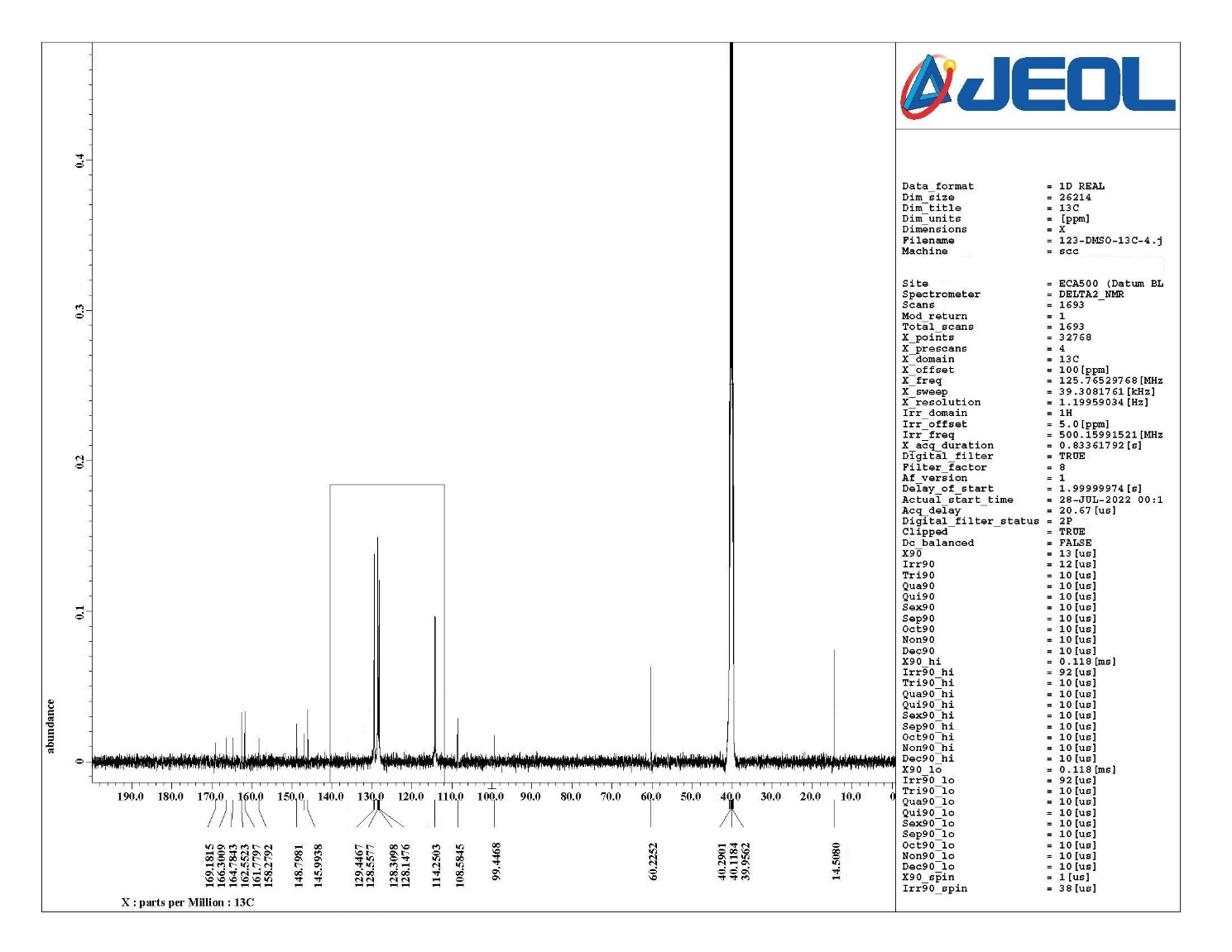
**

**Figure S2: ^13^C-NMR spectrum of compound 4a.**

**
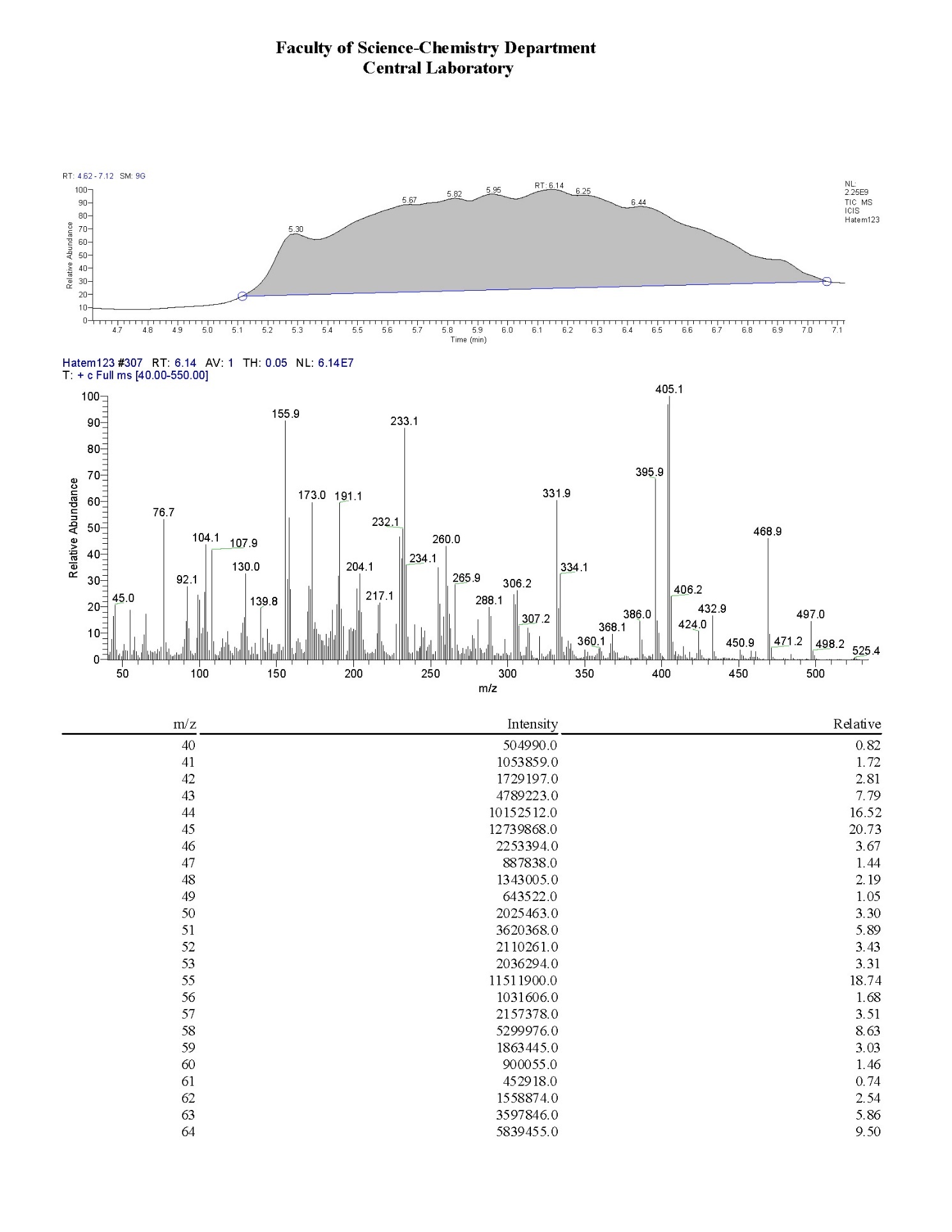
**

**Figure S3: Mass analysis of compound 4a.**

**
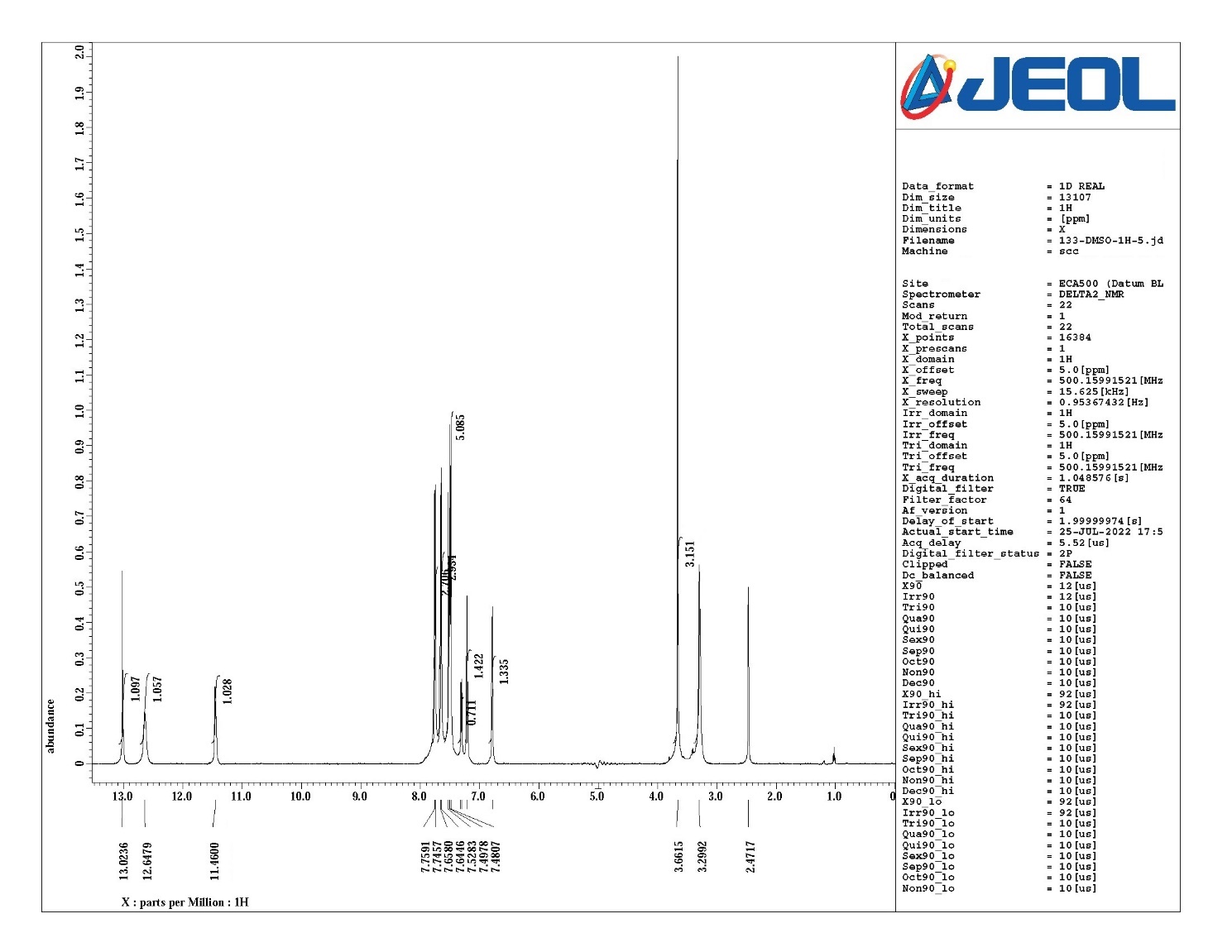
**

**Figure S4: ^1^H-NMR spectrum of compound 4b.**

**
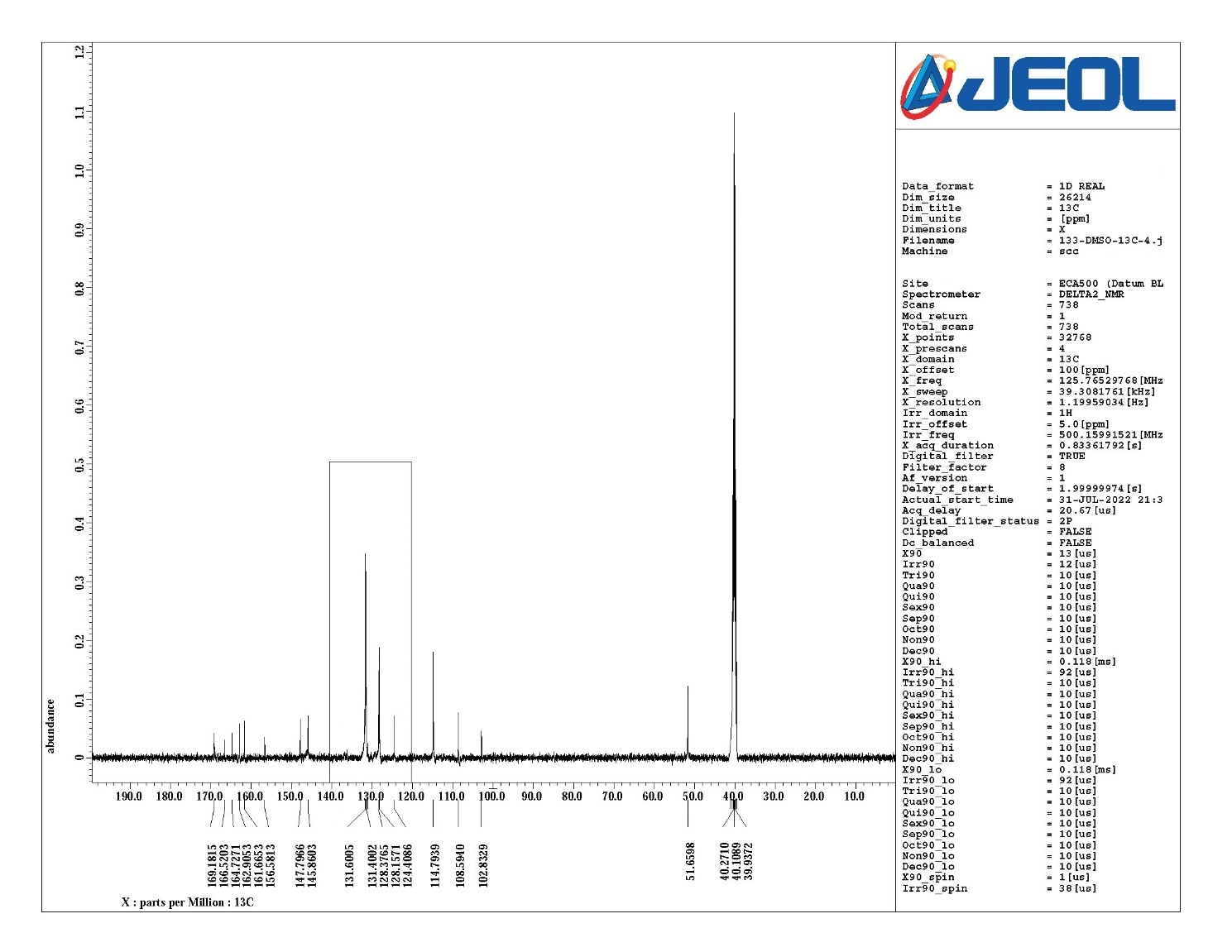
**

**Figure S5: ^13^C-NMR spectrum of compound 4b.**

**
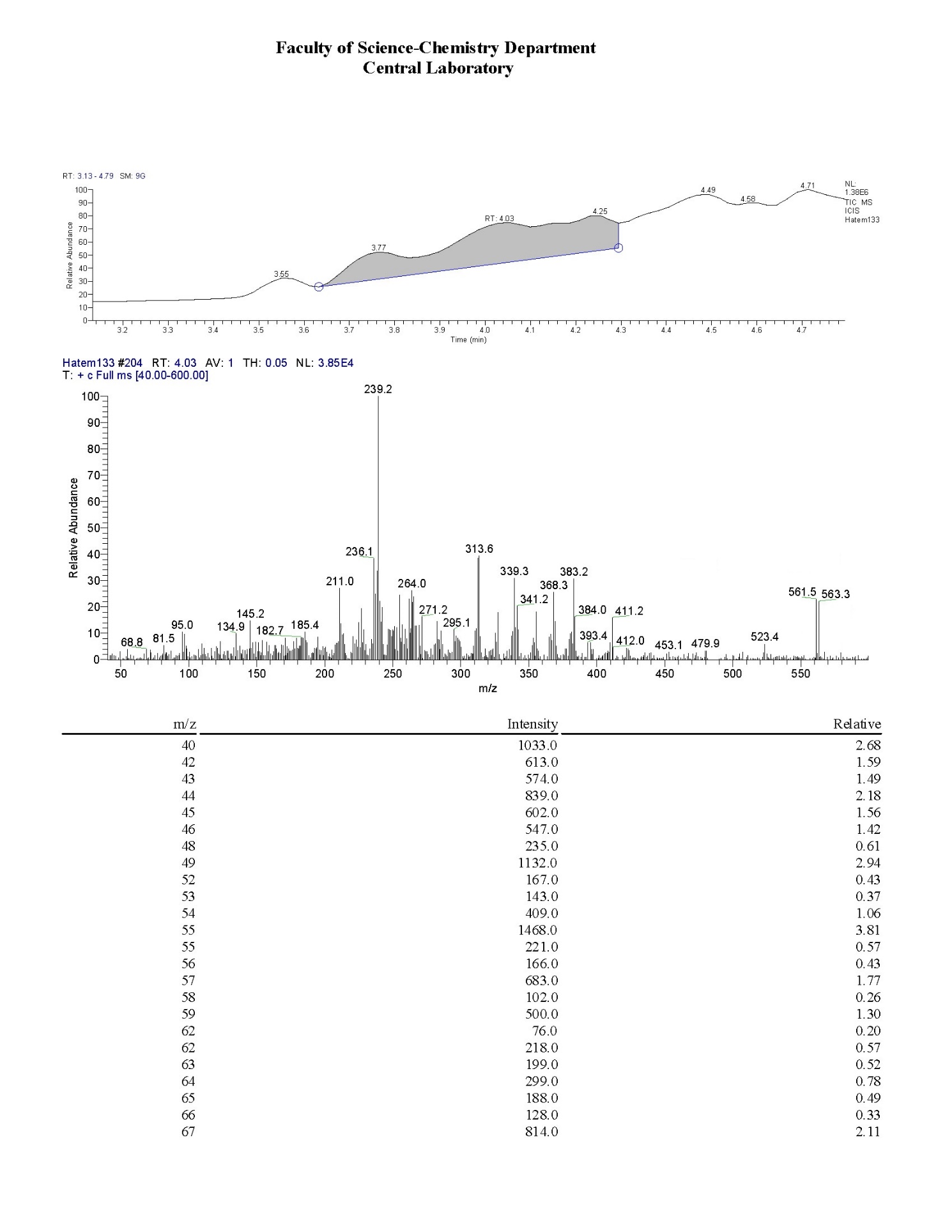
**

**Figure S6: Mass analysis of compound 4b.**

**Figure S7: ^1^H-NMR spectrum of compound 4c.**

**Figure S8: ^13^C-NMR spectrum of compound 4c.**

**
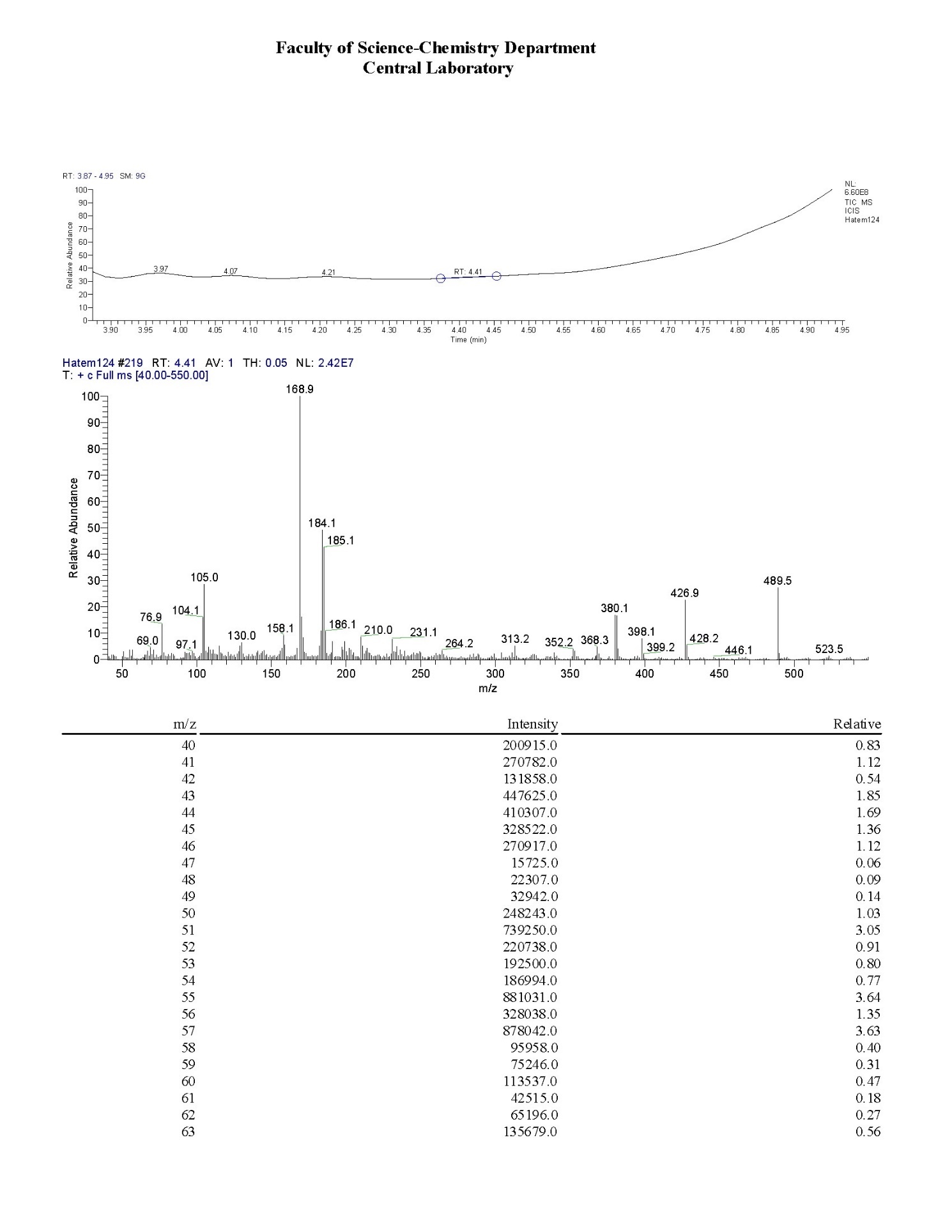
**

**Figure S9: Mass analysis compound 4c.**

**
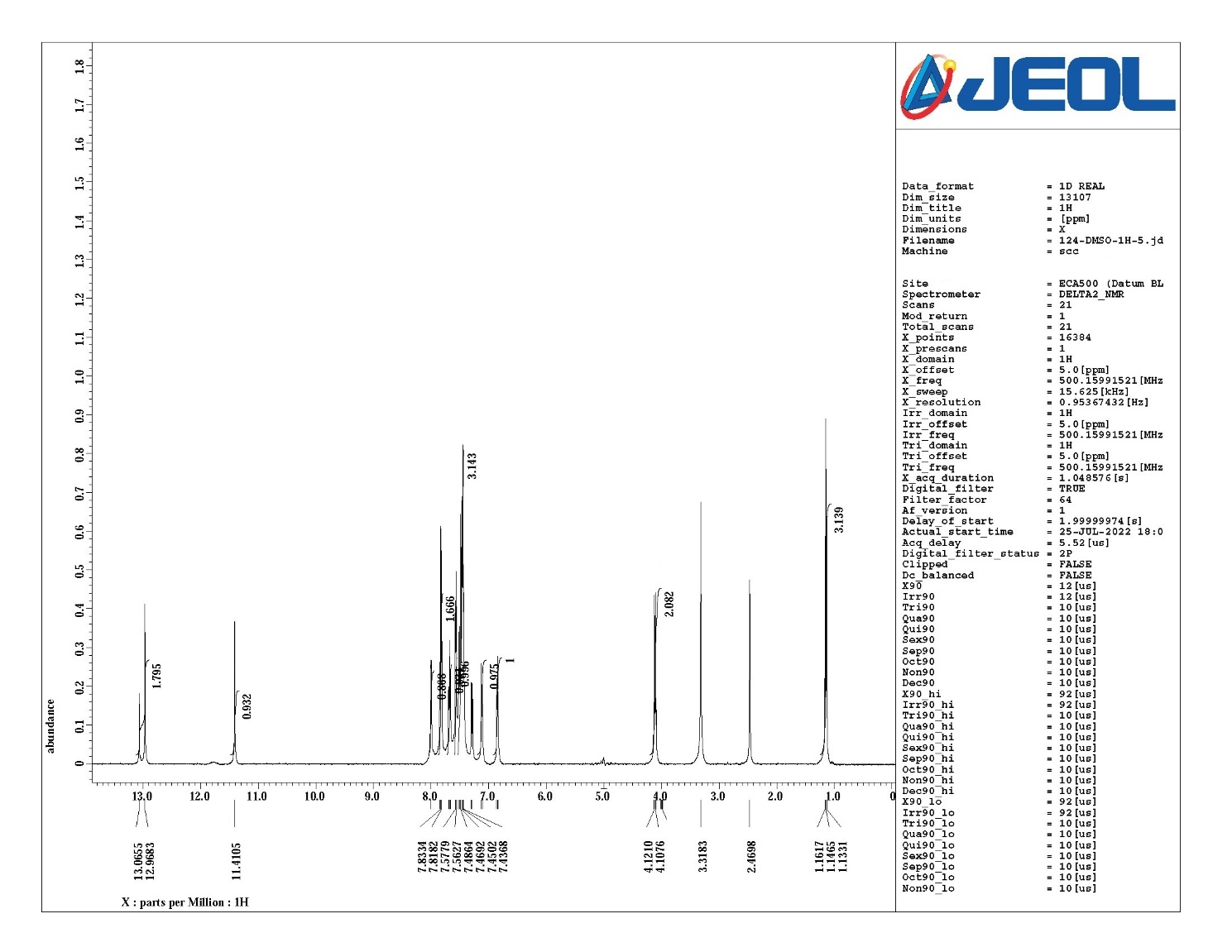
**

**Figure S10: ^1^H-NMR spectrum of compound 4d.**

**
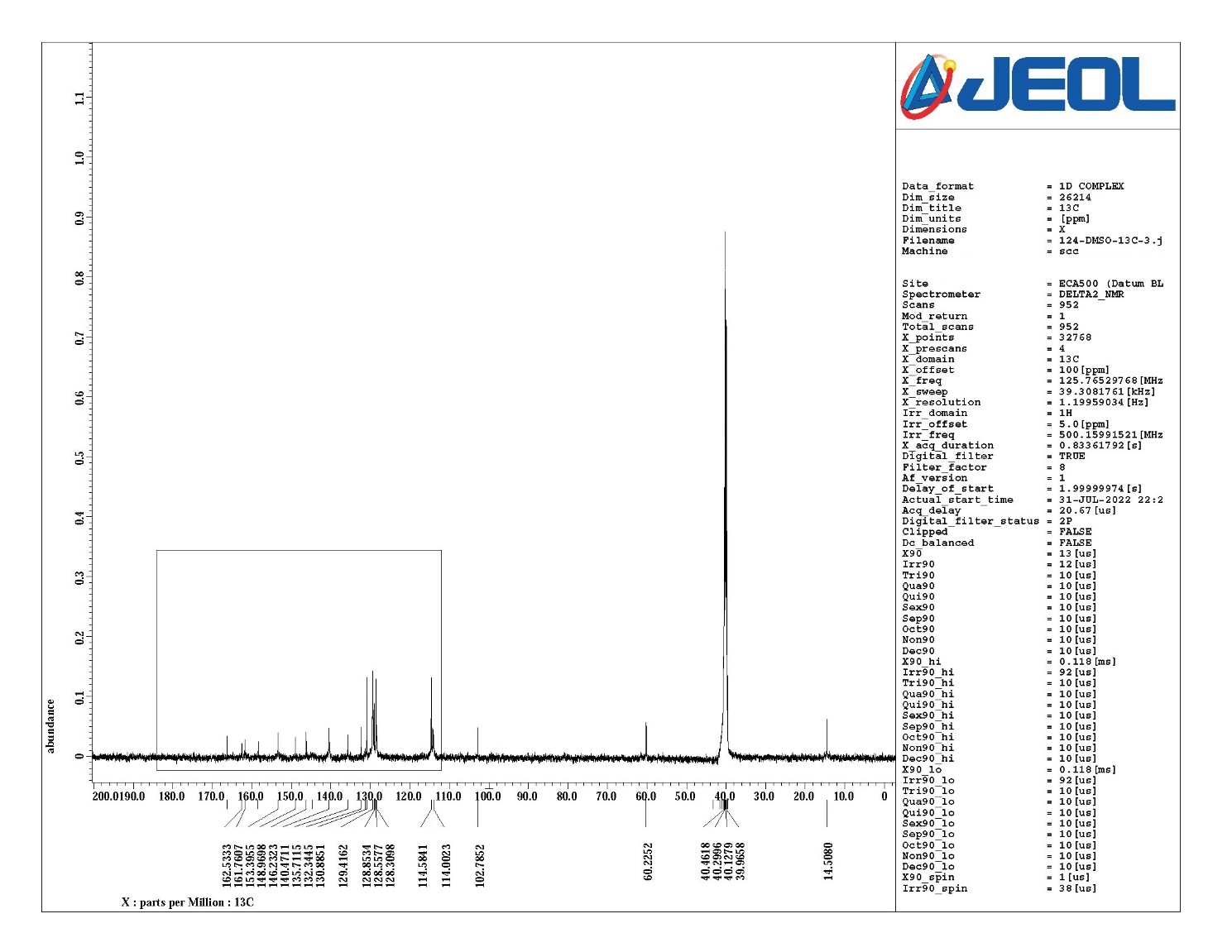
**

**Figure S11: ^13^C-NMR spectrum of compound 4d.**

**
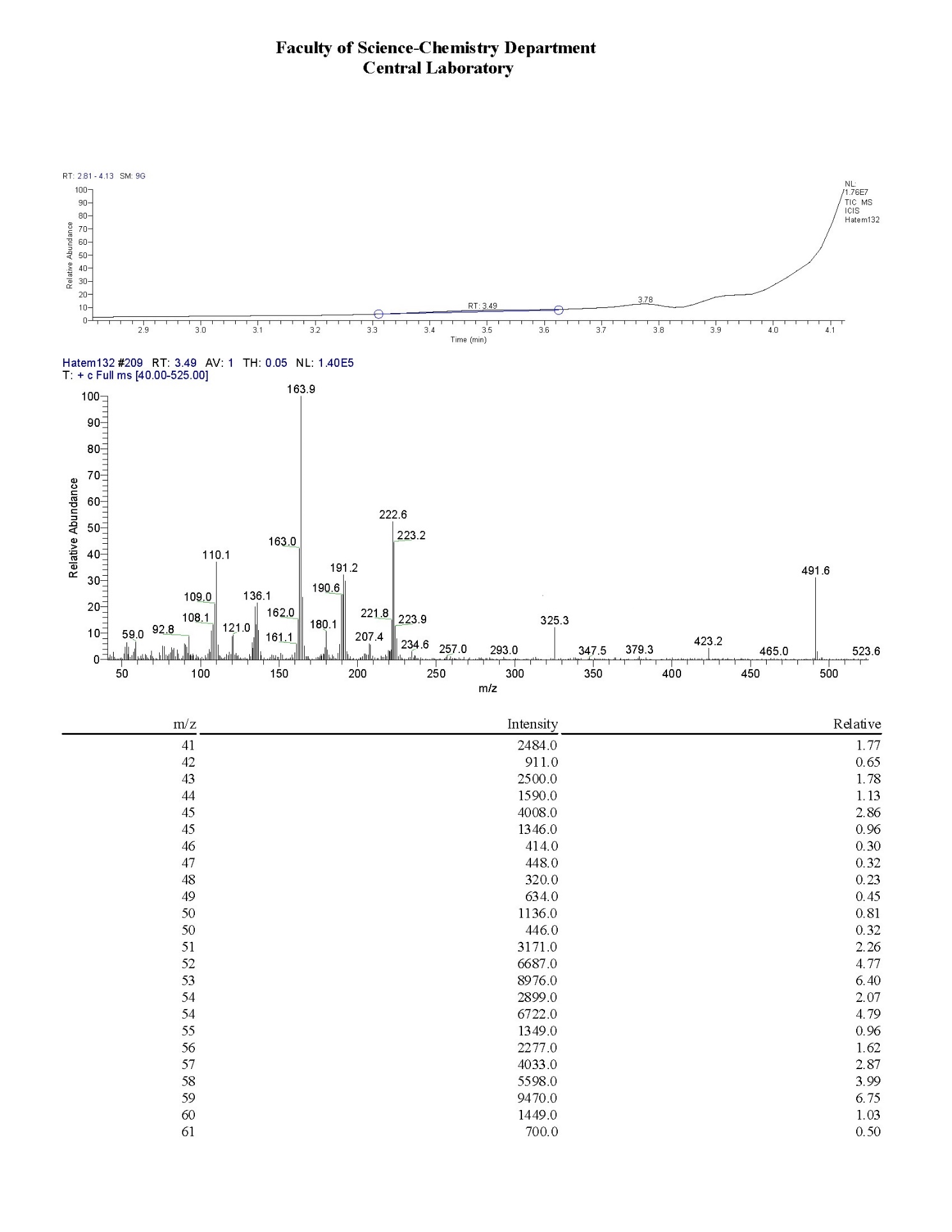
**

**Figure S12: Mass analysis of compound 4d.**

**Figure S13: ^1^H-NMR spectrum of compound 4e.**

**Figure S14: ^13^C-NMR spectrum of compound 4e.**

**
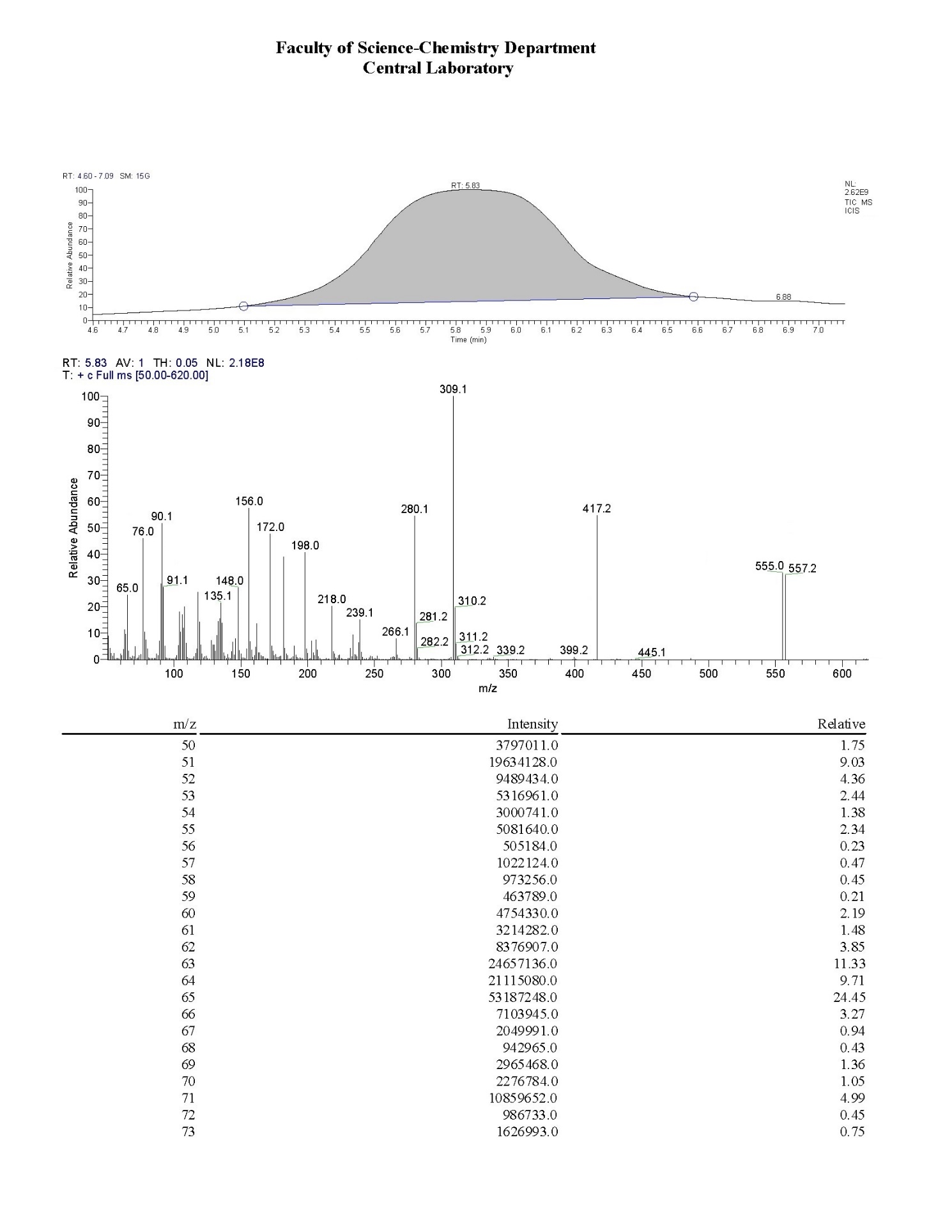
**

**Figure S15: Mass analysis of compound 4e.**

**Figure S16: ^1^H-NMR spectrum of compound 4f.**

**Figure S17: ^13^C-NMR spectrum of compound 4f.**

**
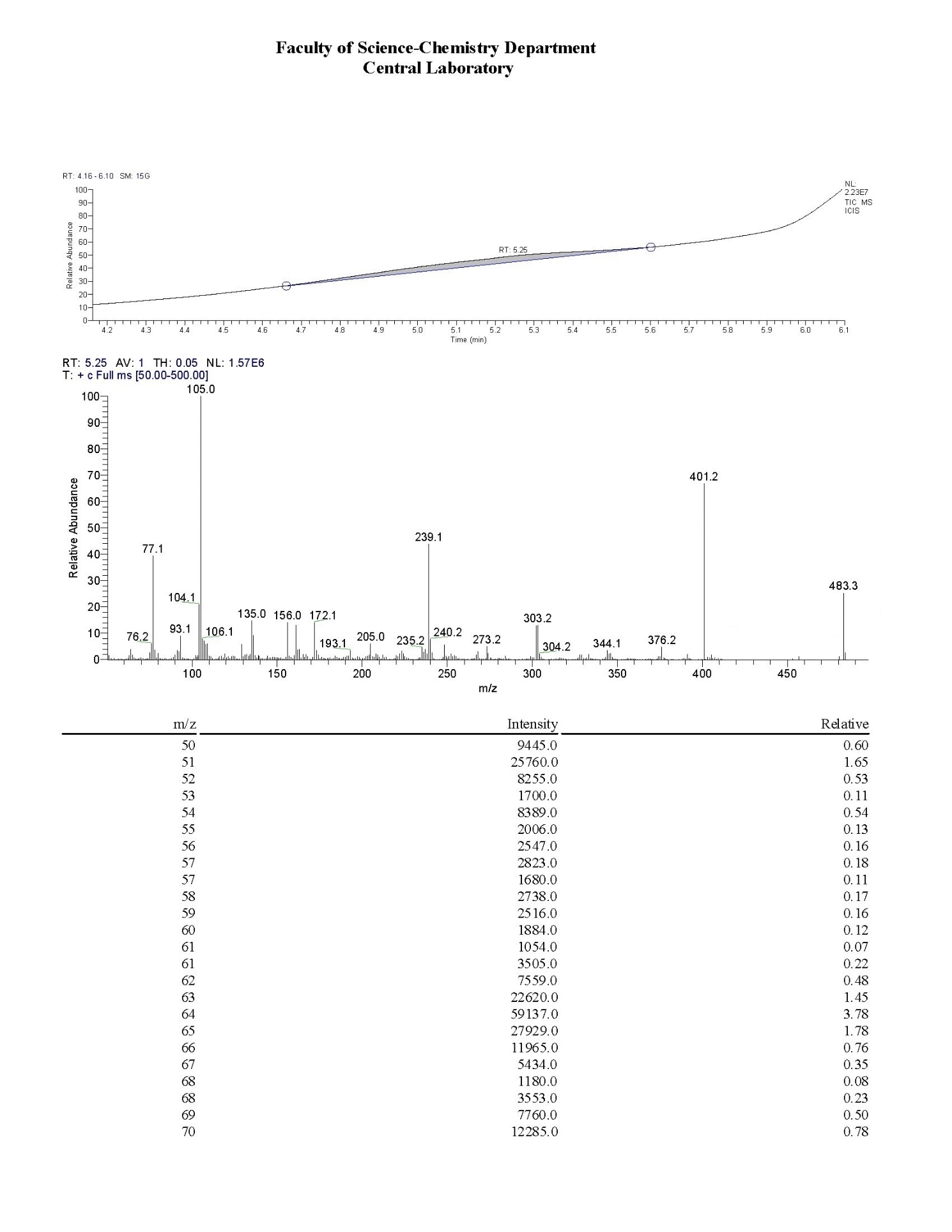
**

**Figure S18: Mass analysis of compound 4f.**
